# Long-Term Persistence of Arbuscular Mycorrhizal Fungi in the Rhizosphere and Bulk Soils of Non-host *Brassica napus* and Their Networks of Co-occurring Microbes

**DOI:** 10.3389/fpls.2022.828145

**Published:** 2022-02-25

**Authors:** Jean-Baptiste Floc’h, Chantal Hamel, Mario Laterrière, Breanne Tidemann, Marc St-Arnaud, Mohamed Hijri

**Affiliations:** ^1^Département de Sciences Biologiques, Institut de Recherche en Biologie Végétale, Université de Montréal, Montréal, QC, Canada; ^2^Quebec Research and Development Centre, Agriculture and Agri-Food Canada, Québec, QC, Canada; ^3^Lacombe Research and Development Centre, Agriculture and Agri-Food Canada, Lacombe, AB, Canada; ^4^African Genome Center, Mohammed VI Polytechnic University (UM6P), Ben Guerir, Morocco

**Keywords:** arbuscular mycorrhizal fungi, rhizosphere, canola, microbiome, crop rotation, amplicon sequencing

## Abstract

Arbuscular mycorrhizal fungi (AMF) are obligate plant symbionts that improve the nutrition and health of their host. Most, but not all the crops form a symbiosis with AMF. It is the case for canola (*Brassica napus*), an important crop in the Canadian Prairies that is known to not form this association. From 2008 to 2018, an experiment was replicated at three locations of the Canadian Prairies and it was used to assess the impact of canola on the community of AMF naturally occurring in three cropping systems, canola monoculture, or canola in two different rotation systems (2-years, canola-wheat and 3-years, barley-pea-canola). We sampled canola rhizosphere and bulk soils to: (i) determine diversity and community structure of AMF, we expected that canola will negatively impact AMF communities in function of its frequency in crop rotations and (ii) wanted to assess how these AMF communities interact with other fungi and bacteria. We detected 49 AMF amplicon sequence variants (ASVs) in canola rhizosphere and bulk soils, confirming the persistence of a diversified AMF community in canola-planted soil, even after 10 years of canola monoculture, which was unexpected considering that canola is among non-mycorrhizal plants. Network analysis revealed a broad range of potential interactions between canola-associated AMF and some fungal and bacterial taxa. We report for the first time that two AMF, *Funneliformis mosseae* and *Rhizophagus iranicus*, shared their bacterial cohort almost entirely in bulk soil. Our results suggest the existence of non-species-specific AMF-bacteria or AMF-fungi relationships that could benefit AMF in absence of host plants. The persistence of an AMF community in canola rhizosphere and bulk soils brings a new light on AMF ecology and leads to new perspectives for further studies about AMF and soil microbes interactions and AMF subsistence without mycotrophic host plants.

## Introduction

Arbuscular mycorrhizal fungi (AMF) are obligate plant symbionts. 400 million years ago, plants were already in association with AMF (Selosse et al., 2015). The arbuscular mycorrhizal symbiosis has evolved with land plants and, as a result, AMF associate with the roots of 90% of the plant species, including most important crops, such as cereals, legumes, and members of Solanaceae (Zhu et al., 2010). The arbuscular mycorrhizal symbiosis brings several benefits for the plant. The AMF facilitate plant nutrition in providing soil nutrients, notably P and N (Smith and Read, 2008) and can mitigate abiotic stresses, such as drought (da Silva Folli-Pereira et al., 2013; Begum et al., 2019), and protect plant roots against soil-borne pathogen attacks (St-Arnaud and Vujanovic, 2007; Lewandowski et al., 2013; Del Fabbro and Prati, 2014). The association of crop plants with AMF often increases crop yield (Hijri, 2016; Séry et al., 2016; Buczkowska and Sałata, 2020).

In bulk and rhizosphere soils, AMF play an important role in nutrient cycling and in structuring the microbial communities (Smith and Smith, 2011; Iffis et al., 2016; Dagher et al., 2020). AMF interact with the other members of the plant and soil microbiome in various ways. With their hyphae, AMF can be viewed as the bacterial highway of the soil: the labile interface between the hyphae and the soil facilitates bacterial migration (Abeysinghe et al., 2020; de Novais et al., 2020). AMF can also interact with phosphorus solubilizing bacteria (Taktek et al., 2017) or can form a tripartite interaction with other fungi to facilitate symbiosis in plant (Liu et al., 2020). But, there is still little knowledge about the interaction of AMF and other microbes in soil and plant rhizosphere.

Certain vascular plants do not host AMF in their roots and it is the case of canola (*Brassica napus*), an important crop in the Canadian Prairies. Canola produces glucosinolates that transform into isocyanates in soil (Smith et al., 2004; Ma et al., 2015). Among others, glucosinolates and isocyanates are toxic, which may explain the non-host plant status of canola. But, from an analysis of canola-associated soil fungi based on fungal internal transcribed spacer (ITS), Floc’h et al. (2021) reported the presence of AMF from the Glomeraceae family in the rhizosphere and bulk soil samples from fields that were in canola monoculture for 10 years. The AMF depend on a host for their carbon needs (Rich et al., 2017) and the persistence of AMF for such a long period of time in absence of mycotrophic host plants is unexpected. However, axenic AMF growth is known to be stimulated *in vitro* by coculture with plant growth-promoting bacteria (Abdellatif et al., 2019). Thus, the report of Floc’h et al. (2021) motivated the verification of this hypothesis: We expect that canola negatively impacts the AMF community in function of its frequency in crop rotations. Furthermore, by using network analysis, we will explore the potential interactions that AMF develop with other microbes, such as fungi and bacteria, in the canola rhizosphere and bulk soil.

In this study, we used a long-term assay held by Agriculture and Agri-Food Canada in three locations of the Canadian Prairies to identify the AMF living in canola rhizosphere and bulk soil and the samples we used are those of Floc’h et al. (2021) from which we extracted and amplified the 18S region of AMF ribosomal DNA (rDNA) to only target AMF. We used network analysis to explore the possible interactions of these AMF with the fungi and bacteria also residing in the canola rhizosphere and bulk soil.

## Materials and Methods

A subset of plots from a long-term experiment initiated in 2008 and replicated at three locations in the Canadian Prairies was sampled in 2018. The experiment tested the effects of diversification in canola-based crop rotation systems. The cultivar of canola used in this study is the canola glufosinate-resistant L241C. Glufosinate amendment was 900 g ha^–1^. The three crop diversification treatments used in this study were: (1) monoculture of canola, (2) wheat-canola, and (3) pea-barley-canola (Table 1). These treatments were applied in a randomized complete block design with four blocks. Plot dimension was 3.7 × 15.2 m. All the rotation phases were present each year at each of the three locations where the experiment was replicated, but only the canola phases of the rotation were used in this study.

**TABLE 1:** Results of the Kruskal–Wallis tests of the effect of crop rotation diversification on indices of arbuscular mycorrhizal fungi (AMF) alpha diversity in canola rhizosphere and bulk soil (α = 0.05).

Rhizosphere						

Index	Crop rotation*^a^*	Mean	Sd	χ^2^	Df*^b^*	*p*-value
Shannon	LL	0	0	4.8875	2	0.0868
	W-LL	0.0583	0.0391			
	P-B-LL	0.174	0.1172			
Simpson	LL	0	0	4.8186	2	0.0898
	W-LL	0.0407	0.0273			
	P-B-LL	0.1148	0.0749			
Richness	LL	0.1667	0	4.068	2	0.1308
	W-LL	0.5083	0.0615			
	P-B-LL	0.7917	0.2046			
**Bulk soil**						
Shannon	LL	0.069	0.0503	1.9619	2	0.3749
	W-LL	0.1215	0.0903			
	P-B-LL	0.2271	0.1178			
Simpson	LL	0.0481	0.0351	1.9619	2	0.3749
	W-LL	0.0815	0.0549			
	P-B-LL	0.1519	0.0787			
Richness	LL	0.275	0.0791	3.838	2	0.1468
	W-LL	0.6167	0.152			
	P-B-LL	0.9583	0.1947			

*^a^LL (for Liberty Link, cultivar L241C), Canola monoculture; W-LL, Wheat-Canola rotation; PB-LL, Pea-Barley-Canola rotation.*

*^b^Df, Degree of freedom.*

The three experiment sites were located in three pedoclimatic zones of the canola-producing regions of Canada. Two sites were in Alberta in the sub-humid brown soil zone: one site in Lacombe (latitude 52.5°N, longitude 113.7°W) and the other site in Lethbridge (latitude 49.7°N, longitude 112.8°W) and a third site was in Swift Current Saskatchewan, in the semi-arid brown soil zone (latitude 50.3°N, longitude 107.7°W). The fourth site (Melfort), used in the study by Floc’h et al. (2021), was discarded. Crops were grown according to best management practices, as described in a study by Harker et al. (2015). The growing season at all the sites was characterized by more frequent rain events in July; just before sampling, Lethbridge was drier (Supplementary Figure 1).

Rhizosphere and bulk soil samples were collected at the mid-bloom stage (50% of flowers opened) of canola development. This occurred in the fourth week of July 2018. Three to four plants within each plot were randomly selected and uprooted with a shovel. The shoots were removed and roots were placed in plastic bags and brought to the laboratory on ice in a cooler. The soil tightly attached to roots was considered as rhizosphere soil. About 5 g of rhizosphere soil per plot was collected by gently brushing the roots. The brushed roots were then gently washed with sterile distilled water. Bulk soil was taken from the top 0–7 cm soil layer by using a 2-cm diameter soil probe, exactly in between two plant rows. The top 0–7 cm of the soil is the “plow layer” in the Canadian Prairie agriculture, in contrast to many soils in humid and subhumid climates, where the plow layer is the top 0–15 or 0–20 cm. The samples were kept at 4°C before being shipped on dry ice to the laboratory in Quebec City, Quebec, where they were preserved at −80°C until DNA extraction.

More details on site description, experimental design, and sampling methods are provided in a study by Floc’h et al. (2020a).

### Deoxyribonucleic Acid Extraction and Amplification

As DNA does not persist well in soil (Romanowski et al., 1993; Widmer et al., 1997; Nielsen et al., 2000), using 18S rDNA to target the AMF community in the rhizosphere and bulk soil of canola was the most adequate approach.

The bulk and rhizosphere samples DNA were extracted by using the PowerSoil™ DNA Isolation Kit (Qiagen, Montreal, Quebec, Canada) for the characterization of resident AMF. The manufacturer’s instructions for both kits were followed, except that soil and rhizosphere DNA were eluted in 50 μl. The DNA extraction of each sample was performed in duplicate and the duplicates were pooled. The quantity and quality of the DNA extracts were first verified on 1.5% agarose gel stained with Biotium GelRed^®^ diluted at ratio 1:10,000 (VWR, Montreal, Quebec, Canada), run at 70 V for 60 min, and visualized by using the Gel Documentation System (Bio-Rad Laboratories, Mississauga, Ontario, Canada). The quantity and quality of the DNA extracts were also verified by using the Qubit Fluorometer version 2.0 (Life Technologies, Burlington, Ontario, Canada) and the Qubit double-stranded DNA (dsDNA) HS Assay Kit. DNA extracts were stored at –20°C until use.

A partial sequence of approximately 800 bp of the nuclear 18S small subunit (SSU) ribosomal RNA gene of AMF was first amplified by using the primer pair AML1/AML2 (Lee et al., 2008). The amplification was performed in 20 μl of reaction mixture in triplicate as follows: 1 μl of genomic DNA (gDNA), 200 μM of each deoxynucleoside triphosphate (dNTP), 2 mM of Mg^2+^, 0.8 μM of each primer, and 2.5 U of Q5 High-Fidelity DNA Polymerase NEBNext^®^ Q5 Hot Start HiFi PCR Master Mix (BioLabs, Whitby, Ontario, Canada). The thermocycling conditions were as follows: initial denaturation at 98°C for 30 s, 20 cycles at 98°C for 10 s, 64°C for 30 s, 65°C for 60 s, and final extension performed at 65°C for 5 min. The DNA was amplified in the Biometra TProfessional Thermocycler (Biometra GmbH, Goettingen, Germany, United Kingdom). The three amplicon replicates were pooled and purified by using the QIAquick PCR Purification Kit (Qiagen, Montreal, Quebec, Canada) and eluted in 50 μl of elution buffer. This step is important to prevent interactions between the remaining primers during nested PCR. PCR products were visualized in a GelRed-stained 1.5% agarose gel.

To comply with the sequencing length capacity of the Illumina MiSeq^®^ Reagent Kit version 3 (2 bp × 300 bp), a new primer pair from Dr. F Stefani laboratory (Ottawa Research and Development Centre, 960 Carling Avenue, Ottawa, ON K1A 06C, Canada) yielding a 490-bp length amplicon (including primers) was used to target the V3-V4 region of the nuclear 18S ribosomal RNA (rRNA) gene: nu-SSU-0450-5′ (5′-CGCAAATTACCCAATCCC-3′) and nu-SSU-0899-3′ (5′-ATAAATCCAAGAATTTCACCTC-3′). Primers were named according to the primer nomenclature system of Gargas and DePriest (1996). The number in the primer name refers to the 5′ end position of the primer on the 18S sequence standard of *Saccharomyces cerevisiae* (GenBank accession Z75578). Primers were designed based on the guidelines provided by the Integrated DNA Technologies (IDT Incorporation, San Diego, California, United States). Purified PCR products amplified with AML1/AML2 were used as templates for nested PCR. A 1–3 bp “heterogeneity spacer” was introduced between the 3′ end of the adapter and the 5′ end of the primer pair nu-SSU-0450-5′/nu-SSU-0899-3′ to dampen the effect of the low sequence diversity issue of the MiSeq platform. The amplification reaction mixture was the same as for the first PCR, except for the primer concentration which was 0.5 μM. The thermocycling conditions were as for the first PCR except for the number of cycles which was reduced to 15 and the annealing temperature which was 59°C. The nested PCR was performed in triplicate. Products were verified by electrophoresis on a GelRed-stained 1.5% agarose gel and replicates were pooled.

Library preparation followed the protocol described in a study by Stefani et al. (2020). Briefly, the PCR products from the nested PCR were purified by using the Agencourt AMPure^®^ XP Beads (Beckman Coulter Incorporation, Indianapolis, Indiana, United States), normalized to 1–2 ng/μl with the SequalPrep™ Normalization Plate Kit (Thermo Fisher Scientific), and indexed by using the Nextera Index Kit (Illumina, San Diego, California, United States). Indexed amplicons were then purified and normalized. Purified indexed amplicons were quantified by quantitative PCR (qPCR) by using the LightCycler^®^ 480 System (Roche Molecular Systems Incorporation, Branchburg, New Jersey, United States) with the KAPA Library Quantification Kit for Illumina platforms (KAPA Biosystems, Massachusetts, United States) to determine the volume of each sample to makeup a 1-nM amplicon pool for library preparation. Paired-end sequencing (2 × 300 bp) was carried out by using the Illumina MiSeq^®^ Sequencer for 500 cycles at the *Centre d’expertise et de service Génome Québec* (Montreal, Quebec, Canada).

### Amplicon Sequence Variant Determination and Bioinformatic Pipeline

Bioinformatics used quantitative insights inoto microbial eology 2 (QIIME2) version 2021.4 (Bolyen et al., 2019). The bioinformatic pipeline used for the processing of nu-SSU-0450-5′ and nu-SSU-0899-3′ 18S small subunit rRNA gene sequences was DADA2 version 1.18.0 (Callahan et al., 2016). First, we used Cutadapt version 3.4 to remove the primer part of the nu-SSU-0450-5′ and nu-SSU-0899-3′ RNA gene sequences with “minimum-length” at 50 and “p-error-rate” at 0.1, “—p-times” at 2 and “—p-overlap” at 6. Then, we excluded the sequences with less than 193 bp on the forward sequences and 195 bp on the reverse sequences with the command “—p-trunc-len” with “—p-mas-ee” set to 2, as the base quality of the sequences tended to diminish below that threshold in our data. Next, the Amplicon Sequence Variant (ASV) table was calculated and chimeras were eliminated. A total of 1,905 ASVs were identified by using the naïve Bayesian classifier method on the National Center for Biotechnology Information (NCBI)/nt database. Only the 62 ASVs belonging to the Mucoromycota and to the Glomeromycota or to an unknown order were kept into a phylogenetic analysis conducted to identify with certainty the ASVs that belong to the group AMF.

The phylogenetic tree was constructed from the alignment of the 62 retained ASVs, 144 sequences from Krüger et al. (2012), and 8 sequences from NCBI/nt database with multiple alignment using fast fourrier transform (MAFFT) (default settings) with the software UGENE version 39.0 (Okonechnikov et al., 2012). A maximum-likelihood tree was calculated in RAxML version 8.2.10 (Stamatakis, 2014) *via* cyberinfrastructure for phylogenetic research (CIPRES) (Miller et al., 2011) with bootstrap resampling set to 1,000 and the GTRGAMMA sequence evolutionary model chosen. The ASVs that were not identified as AMF in the tree were removed to produce a tree with 49 AMF ASVs.

The MiSeq sequencing data generated as part of this study are publicly available on Zenodo.^1^

### Data Processing and Statistical Analyses

We were not able to perform PERMANOVA to assess the effect of crop diversification on AMF community structure due to data structure and neither we were able to do ANOVA to test for the effect of crop diversification on AMF alpha diversity: AMF were sometimes not found in samples leading to an empty row in our matrices. We, thus, used discriminant analysis with the software JMP version 16 (Gonzales, 2021) for community structure and replacement from indicator species analysis and the Kruskal–Wallis test for alpha diversity.

As we wanted to know with what microbes AMF could potentially interact with, we used the exact same samples from a study by Floc’h et al. (2021), in which we reused the ITS and 16S ASV tables of this study in our analysis. The protocols of DNA extraction and amplification, sequencing, and bioinformatic processing of ITS and 16S sequences are described in a study by Floc’h et al. (2021).

To assess the interactions between AMF and fungi and between AMF and bacteria, we created a co-occurrence interkingdom network by using the package SpiecEasi version 1.1.0 in R version 4.1.0 (Kurtz et al., 2015). The analysis considered rhizosphere and bulk soil fungal, bacterial, and AMF communities. The input data consisted of the raw abundance matrices of the AMF, ITS, and 16S ASVs. We first filtered the ITS and 16S datasets to remove ASVs with a frequency lower than 20% to avoid rare species. The SpiecEasi run was conducted with the algorithm “mb” with the lambda min ratio set at 10^–2^ and 50 repetitions. We then imported the networks into Cytoscape version 3.8.2 (Smoot et al., 2011) for plotting and used the “organic” layout to draw the networks. Edges were defined as co-occurrences or mutual exclusion based on the positive or negative values of inverse covariance linking the nodes.

## Results

### Raw Sequencing Datasets

The 7,513,787 reads obtained from sequencing were inputted in the pipeline yielding 4,253,351 non-chimeric reads with a mean of 50,635 reads per sample. Rarefaction curves reached saturation for all the samples (Supplementary Figure 2). These reads were assigned to 1,205 ASVs, which were classified into 49 AMF ASV after phylogenetic filtering and clustering (Supplementary Figure 3), totalizing 222,628 reads distributed heterogeneously across our 72 samples.

### Taxonomic Profiles and Crop Rotation Effect on the Arbuscular Mycorrhizal Fungi Communities of Canola Rhizosphere and Bulk Soils

**FIGURE 1:**
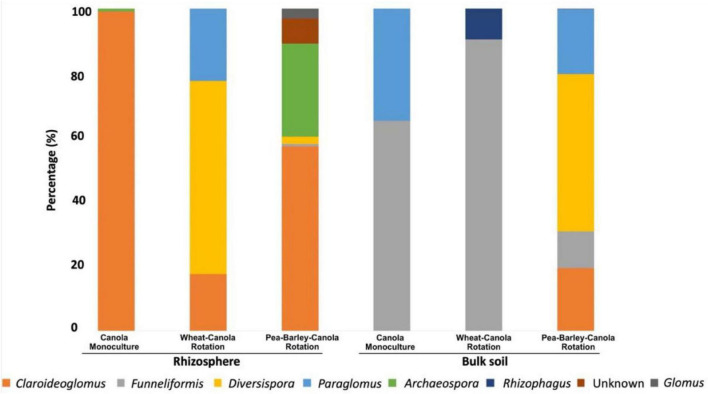
Taxonomic profile of the canola arbuscular mycorrhizal fungi (AMF) community at genus level in function of the crop rotations and biotope. Three degrees of crop rotation systems were used: canola monoculture, wheat-canola rotation, and pea-barley-canola rotation.

The taxonomic profiles of the AMF community differed in canola rhizosphere and bulk soils and varied with crop rotations (Figure 1). However, no significant effect of crop rotations on the alpha diversity of AMF in canola rhizosphere or bulk soil could be detected (Table 1).

In the rhizosphere of monocultured canola, AMF were only represented by two genera: *Claroideoglomus* (99.1%) and *Archaeospora* (0.9%). The 2-crop system (wheat-canola) showed three genera: *Claroideoglomus* (17.7%), *Diversispora* (59.7%), and *Paraglomus* (22.6%). Finally, the 3-crop system (pea, barley, and canola) showed six genera: *Claroideoglomus* (57.2%), *Funneliformis* (0.8%), *Diversispora* (2.2%), *Archaeospora* (28.8%), *Glomus* (3.1%), and one unknown AMF genus (7.7%).

In bulk soil, canola monoculture showed again only two genera of Glomeromycota, *Funneliformis* (65.1%) and *Paraglomus* (34.9%). The 2-crop system (wheat-canola) showed two genera: *Funneliformis* (90.39%) and *Rhizophagus* (9.61%) and the 3-crop system (pea, barley, and canola) showed four genera: *Claroideoglomus* (19.54%), *Funneliformis* (11.42%), *Diversispora* (48.64%), and *Paraglomus* (20.4%).

**FIGURE 2:**
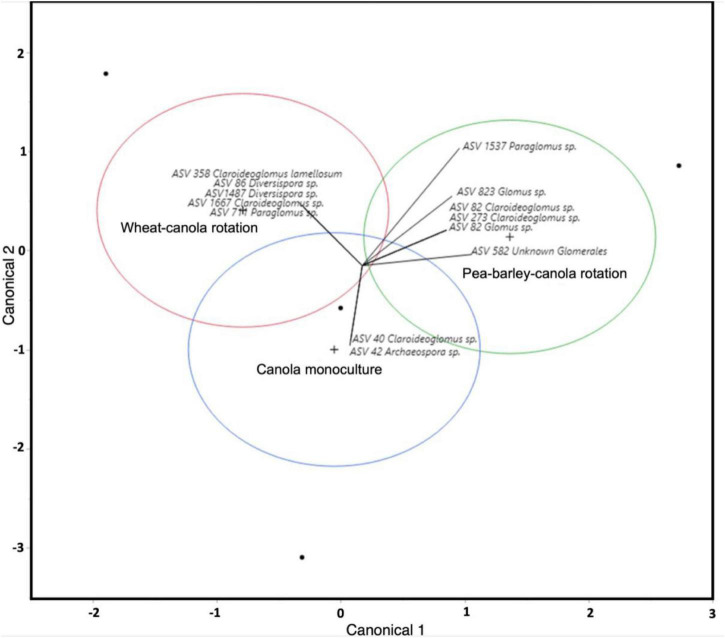
Canonical analysis of AMF community from the canola rhizosphere. Circles in red, blue, and green represent the different crop rotations, while vectors show different AMF amplicon sequence variant (ASV), which are likely to be preferentially associated with each of the crop rotations. The more separated the circles are, the more the community structure between the crop rotations is different. The more the arrow is directed to the center of a circle, the more the ASV was associated with a certain crop rotation.

Crop rotations influenced canola AMF community structure both in the rhizosphere and bulk soil as shown by canonical analyses (Figure 2 and Supplementary Figure 4). In the rhizosphere, two AMF ASV (ASV40 *Claroideoglomus* spp. and ASV42 *Archaeospora* spp.) were associated with canola monoculture, five ASVs (ASV86 and ASV1487 belong to *Diversispora*, ASV358 and ASV1667 belong to *Claroideoglomus*, and ASV711 belongs to *Paraglomus*) were associated with the two-crop rotation system and six ASVs (two *Claroideoglomus*, two *Glomus*, one *Paraglomus*, and an unidentified AMF) were associated with the three-crop rotation system (Supplementary Figure 4).

In bulk soil under canola monoculture, we found ASV149 (*Paraglomus occultum*) and ASV18 [*Funneliformis mosseae* (*F. mosseae*)], under the two-crop system, we found two *Funneliformis*: ASV1254 and ASV10, one *Rhizophagus* ASV1809, and one *Glomus* ASV1396, and under the three-crop system, we found three different *Diversispora*: ASV1774, ASV47, and ASV22, two *Claroideoglomus*: ASV79 and ASV203, and, finally, two *Paraglomus*: ASV61 and ASV1462.

### Network Analysis and the Microbial Cohorts of Canola-Associated Arbuscular Mycorrhizal Fungi

**FIGURE 3:**
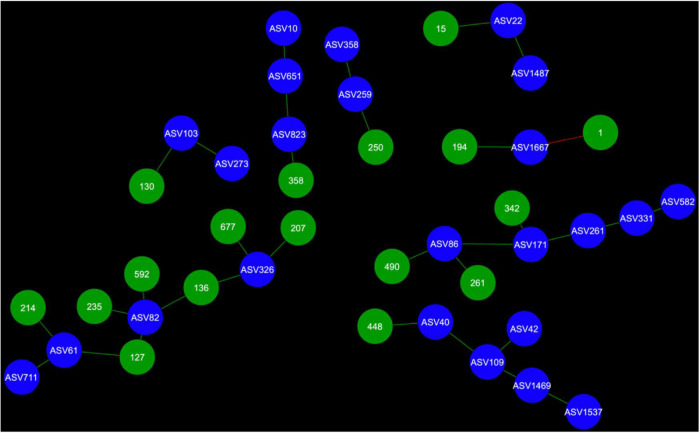
Fungal cohorts of canola AMF in the rhizosphere. The nodes in blue are the AMF ASV and the nodes in green are the fungal ASV (FASV). Red edges signify mutual exclusion, whereas green edges signify positive co-occurrence of species.

**FIGURE 4:**
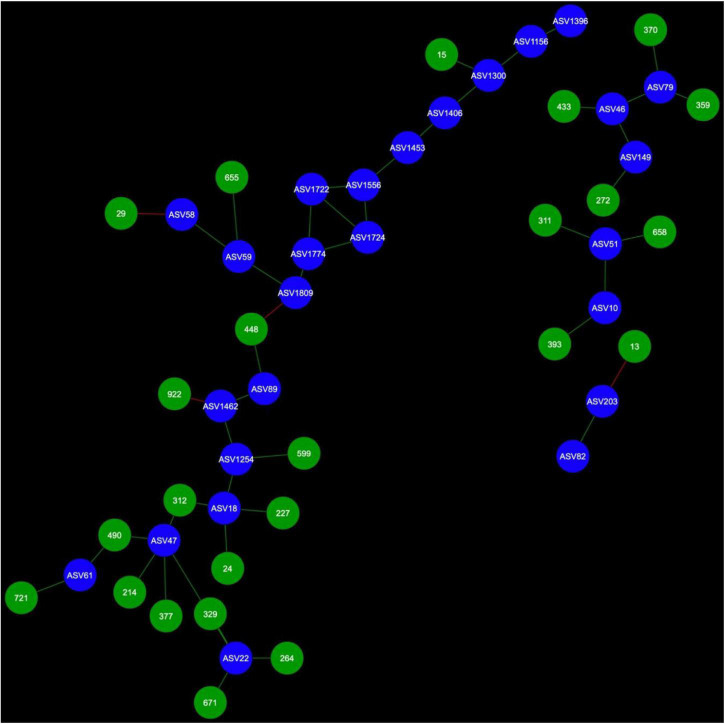
Fungal cohorts of canola AMF in bulk soil. The nodes in blue are the AMF ASV and the nodes in green are the FASV. Red edges signify mutual exclusion, whereas green edges signify positive co-occurrence of species.

**FIGURE 5:**
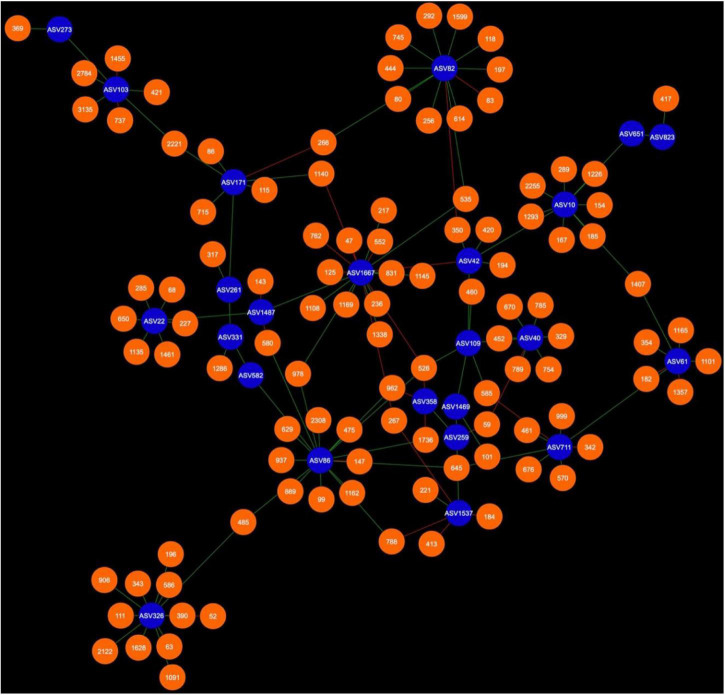
Bacterial cohort of canola AMF in the rhizosphere. The nodes in blue are the AMF ASV and the nodes in orange are the bacterial ASV (BASV). Red edges signify mutual exclusion, whereas green edges signify positive co-occurrence of species.

**FIGURE 6:**
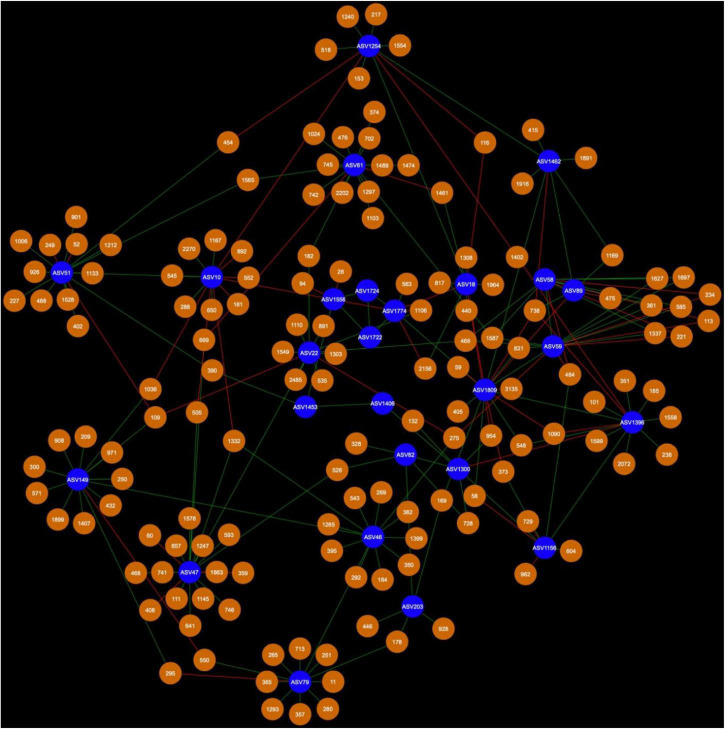
Bacterial cohort of canola AMF in bulk soil. The nodes in blue are the AMF ASV and the nodes in orange are the BASV. Red edges signify mutual exclusion, whereas green edges signify positive co-occurrence of species.

**FIGURE 7:**
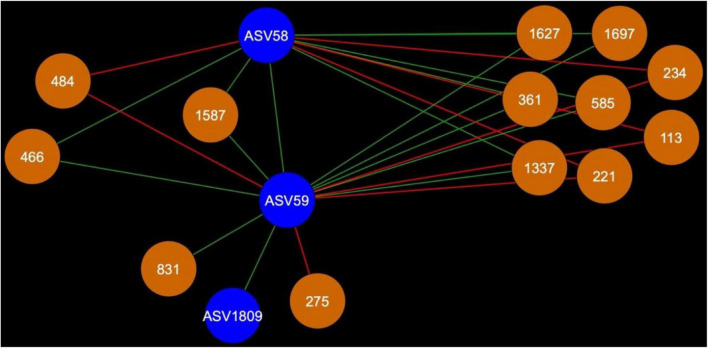
Bacterial cohort shared between AMF ASV58 (*Funneliformis mosseae*) and AMF ASV59 (*Rhizophagus iranicus*). The nodes in blue are the AMF ASV and the nodes in orange are the BASV. Red edges signify mutual exclusion, whereas green edges signify positive co-occurrence of species.

Network analysis revealed that 24 AMF ASVs are putatively interacting in the rhizosphere and 26 AMF ASVs are putatively interacting in the bulk soil (Figures 3–7). Potential interactions between AMF were always co-occurrences, while no mutual exclusions were found.

Microbial networks were broadly different in terms of connectivity. Fungi-AMF networks showed relatively few potential interactions: 41 nodes and 33 edges in the rhizosphere (Figure 3) and 50 nodes and 48 edges in the bulk soil (Figure 4), whereas potential interactions in the bacteria-AMF network were more abundant with 130 nodes and 142 edges in the rhizosphere (Figure 5) and 175 nodes and 227 edges in the bulk soil (Figure 6). The occurrence of AMF ASVs rarely corresponded with the occurrence of fungal ASV (FASV). In contrast, all the AMF ASV, except two (AMF ASV1722 and AMF ASV1724, both identified as *Glomus indicum*), have potential relationships with at least one bacterial ASV (BASV), in each of the interkingdom networks (Figures 5, 6).

In the rhizosphere, AMF cohorts rarely shared common genera with the same type of relationship, but in the bulk soil (Supplementary Table 1), the cohorts were dominated by Vicinamibacteraceae, as this clade was present 21 times across all the AMF cohorts (Supplementary Table 2). In the bulk soil, AMF ASVs rarely shared an important fraction of their bacterial or fungal cohort (Supplementary Tables 1, 2), but AMF ASV58 (*F. mosseae*) and AMF ASV59 [*Rhizophagus iranicus* (*R. iranicus*)] almost entirely shared their microbial cohort (Figure 7). All the BASV shared between the two AMF ASVs were shared with the same type of relationship.

## Discussion

### How an Arbuscular Mycorrhizal Fungi Community Persists After 10 Years of Canola Monoculture?

Arbuscular mycorrhizal fungi depend entirely on a host plant, as they cannot produce palmitic acid by themselves (Trépanier et al., 2005). It is believed that they cannot complete their life cycle, reproduce, and maintain their community in soil without a living host plant. As the experiment was conducted with a canola cultivar possessing the gene of resistance to the herbicide Liberty^®^ and was grown with the Liberty^®^ herbicide technology, weeds could have hardly supported AMF in this study. Weeds were for all-purpose absent, especially in canola plots. Harker et al. (2015) reported weeds 4 years from the onset of this experiment and their weed count, which was done before preseeding burnout, reflects the size of the seed bank at that time. The glufosinate is a powerful non-selective herbicide that killed 90% of the common weeds in North America at a rate of 70 g ha^–1^ (Hoss et al., 2003). In the present experiment, glufosinate was applied at a rate of 900 g ha^–1^; thus, after 10 years, an impact of weeds on the AMF community is very unlikely. Canola, as other Brassicaceae plants, is well documented to be non-mycorrhizal plants (Tommerup, 1984; Anthony et al., 2020) and it was assumed that its successive monoculture leads to a poor presence or absence of AMF in its soil. This study clearly demonstrated the persistence of AMF communities in the soil in which canola was cultivated as a sole crop for a long period of 10 years, even if the community is very poor (Supplementary Table 3). More importantly, we retrieved sequences of several AMF taxa from a narrow zone of influence (rhizosphere) of this presumably non-host crop plant. This could be explained by the following hypotheses:

(i) Arbuscular mycorrhizal fungi may colonize epidermis and external cell layers of canola roots without establishment of functional mycorrhizal symbiosis. Such superficial colonization of canola roots could be sufficient for AMF to undergo limited growth and spore production contributing to their maintenance. Mycorrhizal colonization of the superficial layer of the root cortex of *Arabidopsis thaliana* (*A. thaliana*), another non-host plant of the same family as canola, was previously reported (Veiga et al., 2013; Cosme et al., 2018). AMF can be detrimental to several non-AMF host plant genera, such as *Stelaria* and *Pinus*, by infecting their root interior (Wagg et al., 2011; Veiga et al., 2012); it is possible that AMF could infect canola in a similar way. Fonseca et al. (2001) reported that when *Brassica rapa* was inoculated with AMF, it reacts and increases the concentration of δ15N and δ13C in the shoot, but the influence of AMF in this case is to be relativized as the well-watered regime of the experiment emphasized the dry weight of the plants. Da̧browska et al. (2012) also reported that canola gene *BnMT2* is involved with mycorrhizal symbiosis and changed its expression patterns when canola was inoculated with *Glomus* spores, leading to longer shoots and lower fresh biomass on 10-week-old canola seedlings that suggested a negative effect of the inoculation of AMF spores on canola. As canola roots are not a beneficial environment for AMF, their presence in the canola rhizosphere and soil could be from another reason.

(ii) Bacteria are closely or loosely interacting with AMF mycelia forming biofilms (Seneviratne et al., 2008; Lecomte et al., 2011; Iffis et al., 2014; Guennoc et al., 2017). These biofilms can help the AMF acquire phosphate through phosphate rock solubilization (Taktek et al., 2017) or allow AMF to resist harsh environmental conditions (Iffis et al., 2016). Root endophyte bacteria could be actively recruited by AMF after their penetration into the root interior and have beneficial effects on the plant host (Ujvári et al., 2021). They can also increase AMF root colonization (Bourles et al., 2020; Sangwan and Prasanna, 2021; Ujvári et al., 2021). According to Scheublin et al. (2010) and Agnolucci et al. (2015), the most common culturable bacteria ever identified in the biofilms forming on AMF spores are *Pseudomonas*, *Streptomyces*, *Arthrobacter*, and *Oxalobacteraceae*. In a study by Taktek et al. (2015), the most frequent bacteria encountered were *Burkholderia*; Lecomte et al. (2011) reported the dominance of *Variovorax*, *Bacillus*, *Kocuria*, *Microbacterium*, and *Sphingomonas*; Iffis et al. (2016) reported that *Sphingomonas*, *Pseudomonas*, *Massilia*, and *Methylobacterium* as their most abundant species associated to AMF vesicles in roots. These taxonomic profiles are very different from what we could identify in AMF cohorts by network analysis, as these genera were not detected in this study. With our more inclusive method, we found a high frequency of *Vicinamibacterales* in the cohorts of AMF living in canola rhizosphere and bulk soil, in the Canadian Prairie. *Vicinamibacterales* was associated with tolerance to trace metal contamination of soil, in particular Cu, but its ecological role still remains obscure (Chun et al., 2021). The fact that almost all the AMF in canola rhizosphere and bulk soil had a bacterial cohort in the network analysis comfort the possibility that bacteria could bring advantage to AMF in a hostile environment, facilitating interface with non-host plant. Bacteria acting as host for AMF remains another possibility (Hildebrandt et al., 2006; Horii and Ishii, 2014; Abdellatif et al., 2019).

Arbuscular mycorrhizal fungi may get some palmitate from canola through complex interactions with other microbes, such as mycorrhiza helper bacteria or *Trichoderma* spp. and soil fungi could also be one of the reasons. AMF were able to maintain their presence in canola rhizosphere and bulk soil through the years. However, the number of interactions of AMF with fungi recorded here was much lower than the number of interactions with bacteria (Figures 3–6). It is possible that soil fungus increases yield and overall production of host plants when co-inoculated with AMF and in presence of pathogenic fungi (Martínez-Medina et al., 2009; Nzanza et al., 2012; Commatteo et al., 2019). It can also allow AMF to colonize the rhizosphere of Brassicaceae, such as *A. thaliana* or canola (*B. napus*) (Poveda et al., 2019; Jatana et al., 2021). For example, *Nectria* and *Leptosphaeria* were found in association with AMF spores (Hijri et al., 2002). However, soil fungi ecology remains largely unknown and it is difficult to assess their real ecological roles. The fungi associated with AMF in network analysis could be AMF-helpers candidates, such as FASV592 (unknown Nectriaceae), found in the microbial cohort of AMF ASV82 (*Claroideoglomus* spp.) in the canola rhizosphere. The fact that AMF ASV47 (*Diversispora* spp.) had FASV377 *Olpidium brassicae* in its cohort is also of interest. *O. brassicae* is the most common fungus found in canola roots and its influence on canola is not yet fully understood (Lay et al., 2018a,b; Floc’h et al., 2021). The fact that *O. brassicae* and *Diversispora* spp. in canola bulk soil could share a relationship of co-occurrence is to be noted and cooperation between AMF and *O. brassicae* remains a possibility.

The presence of AMF in canola rhizosphere and bulk soil could also be explained by a combination of these scenarios. However, we do not rule out that dispersion of AMF propagules by wind, rain, and animals may contribute to some extent AMF to persist in canola monoculture overtime (Addy et al., 1998; Smith and Read, 2008; Cornejo et al., 2009; Pepe et al., 2018). Further investigations are needed to shed light on the mechanisms by which AMF persist in soils in the absence of non-host plants. Microbial complexity in soil and plant roots is still poorly understood and microbial interactions in plant microbiomes are just being investigated with mathematical tools since two decades (Friedman et al., 2008; Kurtz et al., 2015). It is also possible that the AMF community retrieved from canola soil and rhizosphere was a remnant of the AMF community of the previous crop from the rotations, other studies report the presence of AMF taxa in the canola rhizosphere (Poveda et al., 2019; Floc’h et al., 2021). Since we found AMF in 10-year-old canola monoculture, AMF coexistence with canola is very likely to be reality.

### Effects of Rotation Systems on Arbuscular Mycorrhizal Fungi Communities and Interactions With Their Associated Microbes

Crop rotation is often used in agriculture to mitigate the accumulation of pathogens that occurs in monoculture and that is the case for canola (Hummel et al., 2009; Harker et al., 2015). Crop rotation is known to be a tool that affects the subterranean microbiota of plants (Suzuki et al., 2012; Xuan et al., 2012; Souza et al., 2013; Detheridge et al., 2016; Fan et al., 2020). However, crop rotation, depending on their diversification, impacts the community structure of canola fungal microbiota, but not its bacterial microbiota in this experiment (Floc’h et al., 2020a,b). Crop rotation is also known to have an impact on the AMF community in host plants (Higo et al., 2014, 2018; Detheridge et al., 2016; Bakhshandeh et al., 2017; Hontoria et al., 2019). In this study, we were not able to test for the effect of crop rotation on AMF community structure due to data scarcity. However, our canonical analysis show a clear differentiation between the AMF community of monoculture, 2-crop, and 3-crop systems (Figure 2 and Supplementary Figure 4). With this, we can hypothesize that AMF community composition, as the composition of fungal community in canola rhizosphere and bulk soil, is influenced by crop rotation diversification.

### Arbuscular Mycorrhizal Fungi ASV58 (*F. mosseae*) and ASV59 (*R. iranicus*) Share Their Microbial Cohort in Canola Bulk Soil

*Funneliformis mosseae* is a widely spread AMF taxon in natural and disturbed environments. It is able to colonize a large range of hosts (Tanwar et al., 2013; Yang et al., 2017; Feitosa de Souza et al., 2018; Jie et al., 2019). It was commonly found in highly polluted environments (Hassan et al., 2011; Li et al., 2020). *Rhizophagus* is also a genus of AMF known for its adaptability and for being an R-strategist (Campagnac and Khasa, 2013; Sêdzielewska Toro and Brachmann, 2016; Buysens et al., 2017; Zhang et al., 2018; Cruz-Paredes et al., 2020; Lee et al., 2020). Members of the *Rhizophagus* genus have been commercialized and utilized as biofertilizers for more than three decades (Badri et al., 2016; Hijri, 2016; Basiru et al., 2021). *Rhizophagus* is also known for its ability to interact with plant growth-promoting bacteria (Battini et al., 2016; Loján et al., 2017). These two AMF ASV seem to share the same ecological niche and interact with the same bacteria. To the best of our knowledge, it is the first time that an overlap of 90% of bacterial cohort between two AMF species is reported. We know very little about the potential ecology and functions of the members shared in *F. mosseae* (AMF ASV58) and *R. iranicus* (AMF ASV59) cohorts. Among 11 taxa shared between AMF ASV58 and AMF ASV59, seven taxa were identified at genus level and among those, only four genera were the subject of more than two scientific publications: *Sphingomonas*, *Altererythrobacter*, *Luteolibacter*, and *Gemmatimonas* (Supplementary Table 2). *Sphingomonas* (BASV234) is the only one with a relation of mutual exclusion with the two AMF ASVs. *Sphingomonas* was isolated from spores of *Rhizophagus irregularis* and showed biofilm-like formation on hyphae (Lecomte et al., 2011). It was also found in leek rhizosphere (Nunes da Rocha et al., 2011) and diverse environments including biological soil crusts and freshwater (Ko et al., 2017; Lee et al., 2017; Zhang et al., 2017). The other three taxa were also found in diverse environments (Jiang et al., 2012; Yuan et al., 2017; Kang et al., 2019; Meng et al., 2019; Dahal et al., 2021) and *Gemmatimonas* was reported as a potential denitrifying bacterium (Chee-Sanford et al., 2019). Potential interactions between *F. mosseae* and *R. iranicus* and the bacterial taxa represented in their cohorts should deserve further research attention.

## Conclusion

A community of AMF can be found in the rhizosphere and bulk soils of canola (*B. napus*), a non-host plant, even after 10 years of canola monoculture. This finding puts a new light on the ecology of AMF in the rhizosphere and bulk soil in absence of a mycotrophic host plant. AMF form a broad range of potential interactions with diverse bacterial and fungal species that may be important for AMF, especially in absence of host plants. How AMF interact with other microbes is unclear, but their associations in network analysis indicate the possibility of a direct or indirect interaction with other fungi and bacteria, which need to be clarified.

## Data Availability Statement

The original contributions presented in the study are publicly available. This data can be found here: The MiSeq sequencing data generated as part of this work are publicly available on Zenodo (https://zenodo.org/record/5639078).

## Author Contributions

J-BF performed the experiment, analyzed the data, and wrote the manuscript with input from all co-authors. BT conducted field experiments and collected the data. CH, MS-A, and MH supervised the work, designed the experiment, obtained fundings, and revised the manuscript. All authors contributed to the article and approved the submitted version.

## Conflict of Interest

The authors declare that the research was conducted in the absence of any commercial or financial relationships that could be construed as a potential conflict of interest.

## Publisher’s Note

All claims expressed in this article are solely those of the authors and do not necessarily represent those of their affiliated organizations, or those of the publisher, the editors and the reviewers. Any product that may be evaluated in this article, or claim that may be made by its manufacturer, is not guaranteed or endorsed by the publisher.

## Footnotes

1
https://zenodo.org/record/5639078
