# Supplementary material for: Long-Term Persistence of Arbuscular Mycorrhizal Fungi in the Rhizosphere and Bulk Soils of Non-host Brassica napus and Their Networks of Co-occurring Microbes
Source: Front Plant Sci. 2022 Feb 25;13:828145. doi: 10.3389/fpls.2022.828145 (PMC8914178; doi:10.3389/fpls.2022.828145)
Supplement: Supplementary file 1 [file Table_1.DOCX]

**Supplementary Information**

**Table S1.**Bacterial and fungal cohort of AMFASVs in the canola rhizosphere. Confidence score is given by RDP classifier and correspond to the probability of correct identification.

**Table S2.**Bacterial and fungal cohort of AMF ASVs in canola bulk soil. Confidence score is given by RDP classifier and correspond to the probability of correct identification.

**Table S3.** Read abundance of each AMF ASV for each site of the experiment.

**Figure S1.**Cumulative precipitations (mm) at each site before sampling in 2018.

**Figure S2.** Phylogenetic tree of the 49 ASV identified as AMF.

**Figure S3.**Canonical analysis of AMF community from the canola rhizosphere. Circles in red, blue and green represent different crop rotations whilevectorsshow different glomeromycotan ASV which are likely to be preferentially associated to each of the crop management. The more separated the circles are, the more the community structure between the crop management are different. The more the arrow is directed to the center of a circle, the more the ASV was associated with a certain crop rotation.

**Figure S4.** Rarefaction curves for each sample before trimming non-AMF ASV.

Table S1. Bacterial and fungal cohort of AMFASVs in the canola rhizosphere. Confidence score is given by RDP classifier and correspond to the probability of correct identification.

| Cohort of ASV1667 (*Claroideoglomus*sp*.*) | | | |
| --- | --- | --- | --- |
| ASV ID | Identification | Confidence | Interaction Type |
| BASV47 | *Chloroflexi*sp*.* | 100% | Negative |
| BASV125 | *Bacillus* sp*.* | 100% | Negative |
| BASV217 | *Frankiales*sp*.* | 99% | Positive |
| BASV236 | *Vicinamibacteraceae*sp. | 99% | Positive |
| BASV267 | *Ilumatobacteraceae*sp. | 100% | Negative |
| BASV526 | *Phenylobacterium*sp. | 100% | Negative |
| BASV535 | *Sphingomonas*sp. | 99% | Positive |
| BASV552 | *Pseudohongiellaceae*sp. | 99% | Negative |
| BASV762 | *Steroidobacteraceae*sp. | 99% | Negative |
| BASV831 | *Chloroflexi*sp. | 92% | Positive |
| BASV978 | *Ilumatobacteraceae*sp. | 98% | Positive |
| BASV1108 | *Dinghuibacter*sp. | 99% | Positive |
| BASV1140 | *Solirubrobacterales*sp. | 98% | Negative |
| BASV1145 | *Gemmatimonas*sp. | 98% | Positive |
| BASV1169 | *Vicinamibacteraceae*sp. | 99% | Positive |
| BASV1338 | *Tepidisphaerales*sp. | 99% | Positive |
| FASV1 | *Humicola*sp. | 100% | Negative |
| FASV194 | Unknown *Ascomycota* | 100% | Positive |

| Cohort of ASV86 (*Diversispora*sp.) | | | |
| --- | --- | --- | --- |
| ASV ID | Identification | Confidence | Interaction Type |
| BASV99 | *Blastococcus*sp. | 100% | Positive |
| BASV147 | *Altererythrobacter*sp. | 99% | Negative |
| BASV475 | *Brevundimonas*sp. | 94% | Positive |
| BASV485 | Unknown*Latescibacterota* | 74% | Positive |
| BASV526 | *Phenylobacterium*sp. | 100% | Positive |
| BASV580 | *Solirubrobacter*sp. | 100% | Positive |
| BASV629 | *Steroidobacter*sp. | 100% | Positive |
| BASV645 | *Acidimicrobiia IMCC26256* sp. | 100% | Positive |
| BASV788 | *Devosia*sp. | 100% | Positive |
| BASV889 | Unknown*Microbacteriaceae* | 82% | Positive |
| BASV937 | Unknown*Gaiellales* | 84% | Positive |
| BASV962 | Unknown | 72% | positive |
| BASV978 | *Ilumatobacteraceae*sp. | 98% | positive |
| BASV1162 | *Microtrichales*sp. | 98% | positive |
| BASV1736 | *Vicinamibacterales*sp. | 100% | positive |
| BASV2308 | Unknown*Gammaproteobacteria* | 73% | positive |
| FASV261 | Unknown *Hypocreales* | 100% | positive |
| FASV490 | *Pulchromyces*sp. | 92% | positive |

| Cohort of ASV82 (*Claroideoglomus*sp.) | | | |
| --- | --- | --- | --- |
| ASV ID | Identification | confidence | Interaction Type |
| BASV80 | *Nocardioides*sp. | 100% | positive |
| BASV83 | Unknown*Chloroflexi* | 88% | negative |
| BASV118 | Unknown *Actinobacteria* | 75% | positive |
| BASV197 | Unknown *Actinobacteria* | 70% | positive |
| BASV256 | *Lapillicoccus*sp. | 100% | positive |
| BASV266 | *Reyranella*sp. | 98% | positive |
| BASV292 | *Actinobacteria MB-A2-108* | 100% | positive |
| BASV350 | Unknown*Gemmatimonadota* | 72% | negative |
| BASV444 | Unknown*Thermoleophilia* | 89% | positive |
| BASV535 | *Sphingomonas*sp. | 100% | positive |
| BASV614 | Unknown*Blastocatellaceae* | 100% | positive |
| BASV745 | Unknown*Chloroflexi* | 75% | positive |
| BASV1599 | *Sphingobacteriales NS11-12* | 100% | positive |
| FASV127 | Unknown*Sordariomycetes* | 100% | positive |
| FASV136 | *Mortierella*sp. | 100% | positive |
| FASV235 | Unknown *Ascomycota* | 100% | positive |
| FASV592 | Unknown*Nectriaceae* | 95% | positive |

| Cohort of ASV326 (*Glomus sp*.) | | | |
| --- | --- | --- | --- |
| ASV ID | Identification | confidence | Interaction Type |
| BASV52 | *Skermanella*sp. | 100% | negative |
| BASV63 | *Skermanella*sp. | 100% | positive |
| BASV111 | Unknown*Nocardioidaceae* | 88% | positive |
| BASV196 | *Chitinophagaceae*sp. | 99% | positive |
| BASV343 | Unknown *Actinobacteria* | 73% | positive |
| BASV390 | *Solirubrobacterales*sp. | 99% | positive |
| BASV485 | Unknown*Latescibacterota* | 72% | positive |
| BASV586 | *Nocardioidaceae*sp. | 100% | positive |
| BASV906 | Unknown*Verrucomicrobiota* | 82% | positive |
| BASV1091 | Unknown*Chloroflexi* | 70% | positive |
| BASV1628 | *Nocardioides*sp. | 90% | positive |
| BASV2122 | Unknown*Acidobacteria* | 71% | positive |
| FASV136 | *Mortierella*sp. | 100% | positive |
| FASV207 | *Bipolaris*sp. | 100% | positive |
| FASV677 | *Cryptococcus* sp. | 100% | positive |

| Cohort of ASV10 (*Funeliformismosseae*) | | | |
| --- | --- | --- | --- |
| ASV ID | Identification | confidence | Interaction Type |
| BASV154 | *Rubrobacter*sp. | 100% | positive |
| BASV167 | *Solirubrobacter*sp. | 100% | positive |
| BASV185 | *Solirubrobacter*sp. | 100% | positive |
| BASV289 | *Sphingomonas*sp. | 99% | positive |
| BASV1226 | *Actinocorallia*sp. | 100% | positive |
| BASV1293 | *Nitrospira*sp. | 100% | positive |
| BASV1407 | *Arenimonas*sp. | 100% | positive |
| BASV2255 | *Chthoniobacter*sp. | 100% | positive |

| Cohort of ASV711 (*Paraglomus*sp.) | | | |
| --- | --- | --- | --- |
| ASV ID | Identification | confidence | Interaction Type |
| BASV342 | Unknown*Vicinamibacteraceae* | 100% | negative |
| BASV461 | *Devosia*sp. | 100% | positive |
| BASV570 | Unknown*Gammaproteobacteria* | 70% | positive |
| BASV585 | *Galbitalea*sp. | 100% | negative |
| BASV645 | *Actinobacteria IMCC26256* | 100% | positive |
| BASV676 | *Nocardioides*sp. | 100% | positive |
| BASV999 | Unknown*Pedosphaeraceae* | 91% | positive |

| Cohort of ASV61 (*Paraglomus sp*.) | | | |
| --- | --- | --- | --- |
| ASV ID | Identification | confidence | Interaction Type |
| BASV182 | *Iamia*sp. | 98% | negative |
| BASV354 | *Bryobacter*sp. | 100% | positive |
| BASV1101 | Unknown*Gemmatimonadaceae* | 99% | positive |
| BASV1165 | *Actinobacteria IMCC26256* | 100% | positive |
| BASV1357 | *Haliangium*sp. | 100% | negative |
| BASV1407 | *Arenimonas*sp. | 99% | positive |
| FASV127 | Unknown*Sordariomycetes* | 100% | positive |
| FASV214 | *Podospora*sp. | 100% | positive |

| Cohort of ASV22 (*Diversispora*sp.) | | | |
| --- | --- | --- | --- |
| ASV ID | Identification | confidence | Interaction Type |
| BASV68 | *Serratia* sp. | 97% | positive |
| BASV227 | *Nocardioides*sp. | 100% | positive |
| BASV285 | *Flavisolibacter*sp. | 78% | positive |
| BASV650 | *Opitutus*sp. | 100% | positive |
| BASV1135 | *Actinobacter MB-A2-108* | 100% | positive |
| BASV1461 | Unknown*Vicinamibacteria* | 75% | positive |
| FASV15 | *Chaetomium* sp. | 100% | positive |

| Cohort of ASV171 (*Claroideoglomus*sp.) | | | |
| --- | --- | --- | --- |
| ASV ID | Identification | confidence | Interaction Type |
| BASV86 | *Acinetobacter* sp. | 99% | positive |
| BASV115 | *Gaiella*sp. | 96% | positive |
| BASV266 | *Reyranella*sp. | 99% | negative |
| BASV715 | Unknown*Chloroflexi* | 71% | positive |
| BASV1140 | *Actinobacter 67-14* | 100% | positive |
| BASV2221 | *BIrii41* sp. | 100% | positive |
| FASV342 | *Stachybotrys*sp. | 100% | positive |

| Cohort of ASV103 (*Claroideoglomus sp*.) | | | |
| --- | --- | --- | --- |
| ASV ID | Identification | Confidence | Interaction Type |
| BASV421 | Unknown*Vicinamibacteria* | 94% | positive |
| BASV737 | *Hyphomicrobium*sp. | 100% | negative |
| BASV1455 | *Labrys* sp. | 100% | negative |
| BASV2221 | *BIrii41* sp. | 100% | positive |
| BASV2784 | *Bacteroidota AKYH767* | 100% | positive |
| BASV3135 | *Xanthomonas* sp. | 100% | positive |
| FASV130 | *Sistotrema*sp. | 100% | positive |

| Cohort of ASV1537 (*Paraglomus*sp.) | | | |
| --- | --- | --- | --- |
| ASV ID | Identification | confidence | Interaction Type |
| BASV184 | Unknown*Solirubrobacteraceae* | 80% | negative |
| BASV221 | Unknown*Acetobacteraceae* | 99% | positive |
| BASV267 | Unknown*Ilumatobacteraceae* | 100% | negative |
| BASV413 | *Actinobacter 67-14* | 100% | negative |
| BASV788 | *Devosia*sp. | 99% | negative |

| Cohort of ASV109 (*Claroideoglomus*sp.) | | | |
| --- | --- | --- | --- |
| ASV ID | Identification | confidence | Interaction Type |
| BASV460 | *Chloroflexi KD4-96* | 98% | positive |
| BASV526 | *Phenylobacteriumsp* | 100% | positive |
| BASV585 | *Galbitalea*sp. | 100% | positive |

| Cohort of ASV1469 (*Archaeospora*sp.) | | | |
| --- | --- | --- | --- |
| ASV ID | Identification | confidence | Interaction Type |
| BASV101 | Unknown*Intrasporangiaceae* | 99% | positive |

| Cohort of ASV259 (*Claroideoglomus*sp.) | | | | | | |  |
| --- | --- | --- | --- | --- | --- | --- | --- |
| ASV ID | Identification | | | Confidence | | Interaction Type |  |
| BASV59 | Unknown*Sphingomonadaceae* | | | 100% | | negative |  |
| FASV250 | *Preussia*sp. | | | 100% | | positive |  |
| Cohort of ASV261 (*Claroideoglomuslamellosum*) | | | | | | | |
| ASV ID | | Identification | confidence | | Interaction Type | | |
| BASV317 | | *Mycobacterium* sp. | 97% | | positive | | |

| Cohort of ASV273 (*Claroideoglomus sp*.) | | | |
| --- | --- | --- | --- |
| ASV ID | Identification | confidence | Interaction Type |
| BASV369 | *Flavisolibacter*sp. | 99% | positive |

| Cohort of ASV331 (*Claroideoglomus*sp.) | | | |
| --- | --- | --- | --- |
| ASV ID | Identification | Confidence | Interaction Type |
| BASV1286 | Unknown*Gaiellales* | 99% | positive |

| Cohort of ASV823 (*Glomus* sp.) | | | |
| --- | --- | --- | --- |
| ASV ID | Identification | confidence | Interaction Type |
| BASV417 | *Microlunatus*sp. | 83% | positive |
| FASV358 | *Sporormiella*sp. | 100% | positive |

| Cohort of ASV40 (*Claroideoglomus sp*.) | | | |
| --- | --- | --- | --- |
| ASV ID | Identification | Confidence | Interaction Type |
| FASV448 | *Mortierella*sp. | 100% | positive |

Table S2. Bacterial and fungal cohort of AMF ASVs in canola bulk soil. Confidence score is given by RDP classifier and correspond to the probability of correct identification.

| Cohort of ASV47 (*Diversisporasp*.) | | | |
| --- | --- | --- | --- |
| ASV ID | Identification | confidence | Interaction Type |
| BASV60 | *Bacillus* sp. | 100% | negative |
| BASV111 | *Kribbella*sp. | 88% | positive |
| BASV359 | *Rubrobacter*sp. | 99% | negative |
| BASV408 | *Cellulomonas* sp. | 98% | negative |
| BASV468 | *CandidatusAlysiosphaera*sp. | 99% | positive |
| BASV505 | Unknown*Chitinophagaceae* | 99% | positive |
| BASV526 | *Phenylobacterium*sp. | 100% | positive |
| BASV593 | Unknown*Vicinamibacterales* | 99% | positive |
| BASV641 | *Actinobacteria 67-14* | 91% | positive |
| BASV657 | *Actinobacteria 67-15* | 99% | positive |
| BASV669 | Unknown*Vicinamibacteraceae* | 99% | positive |
| BASV741 | Unknown*Gemmatimonadaceae* | 100% | positive |
| BASV746 | *Gemmatimonas*sp. | 99% | positive |
| BASV1145 | *Gemmatimonas*sp. | 98% | positive |
| BASV1247 | *Actinobacteria IMCC26256* | 100% | positive |
| BASV1332 | *Bacteria WS2* | 100% | positive |
| BASV1578 | *Gemmatimonadota S0134 terrestrial group* | 97% | positive |
| BASV1863 | Unknown*Gemmatimonadaceae* | 90% | positive |
| FASV214 | *Podospora*sp. | 99% | positive |
| FASV312 | *Podospora*sp. | 100% | positive |
| FASV329 | *Mortierella*sp. | 100% | positive |
| FASV377 | *Olpidiumbrassicae* | 100% | positive |
| FASV490 | *Pulchromyces*sp. | 99% | positive |

| Cohort of ASV61 (*Paraglomus sp*.) | | | |
| --- | --- | --- | --- |
| ASV ID | Identification | confidence | Interaction Type |
| BASV182 | *Iamia*sp. | 98% | positive |
| BASV374 | *Cryobacterium*sp. | 99% | positive |
| BASV476 | Unknown*Gaiellales* | 100% | positive |
| BASV669 | Unknown*Vicinamibacteraceae* | 100% | negative |
| BASV702 | Unknown*Ilumatobacteraceae* | 100% | positive |
| BASV742 | Unknown*Vicinamibacterales* | 72% | positive |
| BASV745 | Unknown*Choloflexi* | 76% | positive |
| BASV1024 | *Altererythrobacter*sp. | 99% | positive |
| BASV1103 | *Chthoniobacter*sp. | 99% | positive |
| BASV1297 | Unknown*Saprospiraceae* | 99% | positive |
| BASV1461 | Unknown*Vicinamibacteraceae* | 100% | negative |
| BASV1474 | Unknown*Burkholderiales* | 70% | positive |
| BASV1489 | Unknown*Pirellulaceae* | 97% | positive |
| BASV1565 | Unknown*Vicinamibacteraceae* | 100% | positive |
| BASV1587 | *Roseisolibacter*sp. | 100% | positive |
| BASV2202 | *Planctomycetota WD2101 soil group* | 97% | positive |
| FASV490 | *Pulchromyces*sp. | 99% | positive |
| FASV721 | Unknown*Pleosporales* | 100% | positive |

| Cohort of ASV51 (*Funneliformismosseae*) | | | |
| --- | --- | --- | --- |
| ASV ID | Identification | confidence | Interaction Type |
| BASV52 | *Skermanella*sp. | 100% | negative |
| BASV109 | Unknown*Vicinamibacteraceae* | 99% | negative |
| BASV227 | *Nocardioides*sp. | 100% | positive |
| BASV249 | *Gaiella*sp. | 100% | negative |
| BASV390 | *Actinobacteriota 67-14* | 100% | positive |
| BASV402 | *Sphingomonas*sp. | 100% | positive |
| BASV454 | *Chloroflexi OLB14* | 100% | positive |
| BASV488 | Unknown*Vicinamibacterales* | 99% | positive |
| BASV901 | *UknownActinobacteriota* | 95% | positive |
| BASV926 | *Nocardioides*sp. | 100% | positive |
| BASV1006 | *Rubellimicrobium*sp. | 95% | positive |
| BASV1133 | *Chloroflexi KD4-96* | 100% | positive |
| BASV1212 | *Nocardia* sp. | 99% | positive |
| BASV1528 | *Actinobacteriota 67-14* | 98% | positive |
| BASV1565 | Unknown*Vicinamibacteraceae* | 100% | positive |
| FASV311 | Unknown*Pleosporales* | 100% | positive |
| FASV658 | Unknown*Sporidiobolales* | 98% | positive |

| Cohort of ASV149 (*Paraglomusoccultum*) | | | |
| --- | --- | --- | --- |
| ASV ID | Identification | confidence | Interaction Type |
| BASV109 | Unknown*Vicinamibacteraceae* | 99% | positive |
| BASV209 | *Pseudoxanthomonas*sp. | 85% | positive |
| BASV250 | *Blastococcus*sp. | 93% | positive |
| BASV295 | Unknown*Xanthobacteraceae* | 100% | positive |
| BASV300 | *Solirubrobacter*sp. | 98% | positive |
| BASV432 | *Rhodococcus*sp. | 94% | positive |
| BASV550 | *Chthoniobacter*sp. | 99% | negative |
| BASV571 | *Flavisolibacter*sp. | 100% | positive |
| BASV908 | *Altererythrobacter*sp. | 84% | positive |
| BASV971 | Unknown*Acidimicrobiia* | 100% | positive |
| BASV1036 | Unknown*Gemmatimonadaceae* | 99% | positive |
| BASV1407 | *Arenimonas*sp. | 100% | positive |
| BASV1899 | *Chthoniobacter*sp. | 100% | positive |
| FASV272 | Unknown *Ascomycota* | 100% | positive |

| Cohort of ASV10 (*Funneliformismosseae*) | | | |
| --- | --- | --- | --- |
| ASV ID | Identification | confidence | Interaction Type |
| BASV181 | *Ilumatobacter*sp. | 73% | negative |
| BASV288 | *Altererythrobacter*sp. | 71% | positive |
| BASV505 | Unknown*Chitinophagaceae* | 100% | negative |
| BASV545 | Unknown*Vicinamibacteraceae* | 100% | positive |
| BASV552 | *Gammaproteobacteria Blyi10* | 100% | negative |
| BASV650 | *Opitutus*sp. | 99% | positive |
| BASV892 | *Nocardioides*sp. | 100% | negative |
| BASV1036 | Unknown*Gemmatimonadaceae* | 99% | negative |
| BASV1167 | *Amaricoccus*sp. | 100% | positive |
| BASV1332 | *Bacteria WS2* | 100% | negative |
| BASV2270 | Unknown*Chitinophagaceae* | 99% | positive |
| FASV393 | Unknown*Phaeosphaeriaceae* | 98% | positive |

| Cohort of ASV1396 (*Glomus sp*.) | | | |
| --- | --- | --- | --- |
| ASV ID | Identification | confidence | Interaction Type |
| BASV101 | Unknown*Intrasporangiaceae* | 100% | positive |
| BASV185 | *Solirubrobacter*sp. | 90% | positive |
| BASV238 | *CandidatusUdaeobacter*sp. | 83% | positive |
| BASV351 | *Actinobacteriota IMCC26256* | 76% | positive |
| BASV548 | Unknown*Vicinamibacteria* | 88% | positive |
| BASV738 | Unknown*Gaiellales* | 100% | negative |
| BASV1090 | *Actinobacteriota 67-14* | 96% | negative |
| BASV1402 | *Edaphobaculum*sp. | 82% | negative |
| BASV1558 | Unknown*Gaiellales* | 100% | positive |
| BASV1599 | *Sphingobacteriales NS11-12* | 98% | positive |
| BASV2072 | *Flavisolibacter*sp. | 100% | positive |

| Cohort of ASV59 (*Rhizophagusiranicus*) | | | |
| --- | --- | --- | --- |
| ASV ID | Identification | confidence | Interaction Type |
| BASV113 | Unknown*Xanthomonadaceae* | 100% | negative |
| BASV221 | Unknown*Acetobacteraceae* | 100% | negative |
| BASV234 | *Sphingomonas*sp. | 99% | negative |
| BASV275 | *Chloroflexi KD4-96* | 100% | negative |
| BASV361 | Unknown*Chloroflexi* | 80% | positive |
| BASV466 | Unknown*Vicinamibacteraceae* | 95% | positive |
| BASV484 | *Iamia*sp. | 80% | negative |
| BASV585 | *Galbitalea*sp. | 100% | positive |
| BASV831 | *Chloroflexi TK10* | 92% | positive |
| BASV1337 | *Altererythrobacter*sp. | 100% | positive |
| BASV1587 | *Roseisolibacter*sp. | 99% | positive |
| BASV1627 | *Luteolibacter*sp. | 98% | positive |
| BASV1697 | *Gemmatimonas*sp. | 100% | positive |
| FASV665 | Unknown*Mortierellales* | 100% | positive |

| Cohort of ASV22 (*Diversispora sp*.) | | | |
| --- | --- | --- | --- |
| ASV ID | Identification | confidence | Interaction Type |
| BASV109 | Unknown*Vicinamibacteraceae* | 99% | negative |
| BASV182 | *Iamia*sp. | 98% | positive |
| BASV275 | *Chloroflexi KD4-96* | 100% | negative |
| BASV466 | Unknown*Vicinamibacteraceae* | 95% | positive |
| BASV535 | *Sphingomonas*sp. | 99% | positive |
| BASV891 | *Tepidisphaerales WD2101 soil group* | 100% | positive |
| BASV1106 | *Planctomycetota OM190* | 100% | positive |
| BASV1110 | *Adhaeribacter*sp. | 71% | positive |
| BASV1303 | Unknown*Vicinamibacterales* | 98% | positive |
| BASV1549 | Unknown*Gemmatimonadaceae* | 90% | positive |
| BASV2485 | *Nocardioides*sp. | 95% | positive |
| FASV264 | *Ilyonectria*sp. | 100% | positive |
| FASV329 | *Mortierella*sp. | 100% | positive |
| FASV671 | *Coniophora*sp. | 100% | positive |

| Cohort of ASV46 (*Paraglomusoccultum*) | | | |
| --- | --- | --- | --- |
| ASV ID | Identification | confidence | Interaction Type |
| BASV184 | *Conexibacter*sp. | 80% | positive |
| BASV269 | Unknown*Micromonosporaceae* | 100% | positive |
| BASV275 | *Chloroflexi KD4-96* | 100% | positive |
| BASV292 | *Actinobacteriota MB-A2-108* | 100% | positive |
| BASV350 | *Gemmatimonas*sp. | 78% | positive |
| BASV382 | *Planctomycetota WD2101* | 100% | positive |
| BASV395 | *Acidobacteriota RB41* | 100% | positive |
| BASV543 | *Streptosporangium*sp. | 100% | positive |
| BASV1265 | *Abditibacterium*sp. | 100% | positive |
| BASV1332 | *Bacteria WS2* | 100% | positive |
| BASV1399 | *Gemmatimonas*sp. | 100% | positive |
| FASV433 | *Paecilomyces sp*. | 100% | positive |

| Cohort of ASV58 (*Funneliformismosseae*) | | | |
| --- | --- | --- | --- |
| ASV ID | Identification | confidence | Interaction Type |
| BASV113 | Unknown*Xanthomonadaceae* | 100% | negative |
| BASV221 | Unknown*Acetobacteraceae* | 100% | negative |
| BASV234 | *Sphingomonas*sp. | 99% | negative |
| BASV361 | Unknown*Chloroflexi* | 80% | positive |
| BASV466 | Unknown*Vicinamibacteraceae* | 95% | positive |
| BASV484 | *Iamia*sp. | 80% | negative |
| BASV585 | *Galbitalea*sp. | 100% | positive |
| BASV1337 | *Altererythrobacter*sp. | 100% | positive |
| BASV1587 | *Roseisolibacter*sp. | 99% | positive |
| BASV1627 | *Luteolibacter*sp. | 98% | positive |
| BASV1697 | *Gemmatimonas*sp. | 100% | positive |
| FASV29 | Unknown Ascomycota | 100% | negative |

| Cohort of ASV79 (*Claroideoglomus sp*.) | | | |
| --- | --- | --- | --- |
| ASV ID | Identification | confidence | Interaction Type |
| BASV11 | Unknown*Micrococcaceae* | 100% | positive |
| BASV178 | *Actinobacteriota 67-14* | 92% | positive |
| BASV251 | *Altererythrobacter*sp. | 100% | positive |
| BASV265 | *Bacillus* sp. | 100% | positive |
| BASV280 | Unknown*Chloroflexi* | 70% | positive |
| BASV295 | Unknown*Xanthobacteraceae* | 100% | negative |
| BASV357 | *Actinobacteriota 67-14* | 73% | positive |
| BASV365 | *Rubrobacter*sp. | 100% | positive |
| BASV550 | *Chthoniobacter*sp. | 99% | positive |
| BASV713 | *Luteolibacter*sp. | 94% | positive |
| BASV1293 | *Nitrospira*sp. | 100% | positive |
| FASV359 | *Mortierella*sp. | 100% | positive |
| FASV370 | *Humicola*sp. | 100% | positive |

| Cohort of ASV1809 (*Rhizophagusiranicus*) | | | |
| --- | --- | --- | --- |
| ASV ID | Identification | confidence | Interaction Type |
| BASV58 | Unknown*Intrasporangiaceae* | 99% | positive |
| BASV59 | Unknown*Sphingomonadaceae* | 100% | negative |
| BASV169 | *Iamia*sp. | 86% | positive |
| BASV373 | Unknown*Gaiellales* | 99% | negative |
| BASV405 | *Sphingomonas*sp. | 98% | positive |
| BASV548 | *Acidobacteriota Subgroup 17* | 88% | positive |
| BASV738 | Unknown*Gaiellales* | 98% | negative |
| BASV831 | *Chloroflexi TK10* | 92% | positive |
| BASV3135 | Unknown*Xanthomonadaceae* | 100% | negative |
| FASV448 | *Mortierella*sp. | 100% | negative |

| Cohort of ASV1254 (*Funneliformismosseae*) | | | |
| --- | --- | --- | --- |
| ASV ID | Identification | confidence | Interaction Type |
| BASV116 | *Chryseolinea*sp. | 99% | negative |
| BASV153 | *Ferruginibacter*sp. | 83% | positive |
| BASV217 | *Ferruginibacter*sp. | 99% | positive |
| BASV454 | *Chloroflexi OBL14* | 100% | negative |
| BASV518 | Unknown*Vicinamibacteraceae* | 100% | positive |
| BASV1240 | *Aurantisolimonas*sp. | 99% | positive |
| BASV1402 | *Edaphobaculum*sp. | 82% | negative |
| BASV1554 | *Roseisolibacter*sp. | 100% | positive |
| FASV599 | *Acremonium* sp. | 99% | positive |

| Cohort of ASV18 (*Funneliformismosseae*) | | | |
| --- | --- | --- | --- |
| ASV ID | Identification | confidence | Interaction Type |
| BASV116 | *Chryseolinea*sp. | 99% | negative |
| BASV440 | *Flavisolibacter*sp. | 72% | positive |
| BASV817 | Unknown*Vicinamibacterales* | 100% | positive |
| BASV954 | *Actinobacteriota 67-14* | 94% | negative |
| BASV1308 | *Planctomycetota WD2101* | 100% | positive |
| BASV1461 | Unknown*Vicinamibacteraceae* | 99% | positive |
| BASV1964 | *Edaphobaculum*sp. | 89% | positive |
| BASV2156 | Unknown*Kapabacteriales* | 100% | positive |
| FASV24 | *Mortierella*sp. | 100% | positive |
| FASV227 | *Schizothecium*sp. | 99% | positive |
| FASV312 | *Podospora*sp. | 100% | positive |

| Cohort of ASV1156 (*Glomeraceae sp*.) | | | |
| --- | --- | --- | --- |
| ASV ID | Identification | confidence | Interaction Type |
| BASV58 | Unknown*Intrasporangiaceae* | 99% | negative |
| BASV373 | Unknown*Gaiellales* | 99% | positive |
| BASV484 | *Iamia*sp. | 80% | positive |
| BASV604 | Unknown*Micropepsaceae* | 92% | positive |
| BASV729 | *Qipengyuania*sp. | 100% | negative |
| BASV962 | Unknown*Rhodanobacteraceae* | 72% | negative |

| Cohort of ASV1462 (*Paraglomus sp*.) | | | |
| --- | --- | --- | --- |
| ASV ID | Identification | confidence | Interaction Type |
| BASV415 | *Opitutus*sp. | 99% | positive |
| BASV738 | Unknown*Gaiellales* | 100% | negative |
| BASV1169 | Unknown*Vicinamibacteraceae* | 100% | positive |
| BASV1891 | Unknown*Gammaproteobacteria* | 100% | positive |
| BASV1916 | Unknown*Chitinophagaceae* | 83% | positive |
| FASV922 | *Phialocephala*sp. | 100% | negative |

| Cohort of ASV203 (*Claroideoglomus sp*.) | | | |
| --- | --- | --- | --- |
| ASV ID | Identification | confidence | Interaction Type |
| BASV169 | *Iamia*sp. | 83% | positive |
| BASV178 | *Actinobacteriota 67-14* | 92% | positive |
| BASV446 | *Cellvibrio*sp. | 100% | positive |
| BASV928 | *Pedobacter*sp. | 76% | positive |
| FASV13 | UnknownNectriaceae | 100% | negative |

| Cohort of ASV1300 (*Claroideoglomus sp*.) | | | |
| --- | --- | --- | --- |
| ASV ID | Identification | confidence | Interaction Type |
| BASV132 | Unknown*Gaiellales* | 100% | positive |
| BASV728 | *Ferruginibacter*sp. | 93% | positive |
| BASV954 | *Actinobacteriota 67-14* | 99% | positive |
| FASV15 | *Chaetomium* sp. | 99% | positive |

| Cohort of ASV1774 (*Diversispora*sp.) | | | |
| --- | --- | --- | --- |
| ASV ID | Identification | confidence | Interaction Type |
| BASV563 | *Oceanobacillus*sp. | 93% | positive |
| BASV1106 | *Planctomycetota OM190* | 99% | negative |
| BASV2156 | Unknown*Kapabacteriales* | 100% | negative |

| Cohort of ASV82 (*Claroideoglomus sp*.) | | | |
| --- | --- | --- | --- |
| ASV ID | Identification | confidence | Interaction Type |
| BASV328 | *Porphyrobacter*sp. | 99% | positive |
| BASV526 | *Phenylobacterium*sp. | 99% | positive |
| BASV728 | *Ferruginibacter*sp. | 93% | positive |

| Cohort of ASV89 (*Paraglomusoccultum*) | | | |
| --- | --- | --- | --- |
| ASV ID | Identification | confidence | Interaction Type |
| BASV475 | *Brevundimonas*sp. | 94% | negative |
| BASV738 | Unknown*Gaiellales* | 99% | positive |
| BASV1169 | Unknown*Vicinamibacteraceae* | 100% | positive |
| FASV448 | *Mortierella*sp. | 100% | positive |

| Cohort of ASV1556 (*Glomus indicum*) | | | |
| --- | --- | --- | --- |
| ASV ID | Identification | confidence | Interaction Type |
| BASV28 | *Bradyrhizobium*sp. | 100% | negative |
| BASV94 | *Chloroflexi JG30-KF-CM45* | 100% | negative |

| Cohort of ASV1453 (*Diversispora sp*.) | | | |
| --- | --- | --- | --- |
| ASV ID | Identification | confidence | Interaction Type |
| BASV390 | *Actinobacteriota 67-14* | 100% | positive |

Table S3. Read abundance of each AMF ASV for each site of the experiment.

|  | Rhizosphere | | | Bulksoil | | |
| --- | --- | --- | --- | --- | --- | --- |
| ASV_ID | Lethbridge | Lacombe | Swift Current | Lethbridge | Lacombe | Swift Current |
| ASV10 | 0 | 0 | 310 | 9374 | 35298 | 3887 |
| ASV18 | 0 | 0 | 0 | 10261 | 0 | 12910 |
| ASV22 | 0 | 1840 | 0 | 0 | 17412 | 0 |
| ASV27 | 0 | 0 | 0 | 0 | 0 | 0 |
| ASV40 | 0 | 0 | 11572 | 0 | 0 | 0 |
| ASV42 | 0 | 0 | 10958 | 0 | 0 | 0 |
| ASV46 | 0 | 0 | 0 | 0 | 0 | 5847 |
| ASV47 | 0 | 0 | 0 | 0 | 10028 | 0 |
| ASV51 | 0 | 0 | 0 | 0 | 9488 | 0 |
| ASV58 | 0 | 0 | 0 | 0 | 7685 | 0 |
| ASV59 | 0 | 0 | 0 | 0 | 7615 | 0 |
| ASV61 | 0 | 2017 | 0 | 0 | 5159 | 0 |
| ASV79 | 0 | 0 | 0 | 0 | 0 | 5324 |
| ASV82 | 0 | 1153 | 0 | 0 | 4058 | 10 |
| ASV86 | 0 | 4953 | 0 | 0 | 0 | 0 |
| ASV89 | 0 | 0 | 0 | 0 | 0 | 4687 |
| ASV103 | 0 | 3821 | 0 | 0 | 0 | 0 |
| ASV109 | 0 | 0 | 3510 | 0 | 0 | 0 |
| ASV149 | 0 | 0 | 0 | 0 | 0 | 1212 |
| ASV171 | 0 | 1911 | 0 | 0 | 0 | 0 |
| ASV203 | 0 | 0 | 0 | 0 | 1625 | 0 |
| ASV259 | 0 | 1080 | 0 | 0 | 0 | 0 |
| ASV261 | 0 | 1074 | 0 | 0 | 0 | 0 |
| ASV273 | 0 | 1011 | 0 | 0 | 0 | 0 |
| ASV326 | 0 | 758 | 0 | 0 | 0 | 0 |
| ASV331 | 0 | 745 | 0 | 0 | 0 | 0 |
| ASV358 | 0 | 680 | 0 | 0 | 0 | 0 |
| ASV582 | 0 | 312 | 0 | 0 | 0 | 0 |
| ASV651 | 0 | 0 | 263 | 0 | 0 | 0 |
| ASV711 | 0 | 223 | 0 | 0 | 0 | 0 |
| ASV823 | 0 | 0 | 162 | 0 | 0 | 0 |
| ASV1156 | 0 | 0 | 0 | 0 | 0 | 68 |
| ASV1254 | 0 | 0 | 0 | 24 | 0 | 26 |
| ASV1300 | 0 | 0 | 0 | 0 | 0 | 42 |
| ASV1396 | 0 | 0 | 0 | 0 | 0 | 28 |
| ASV1406 | 0 | 0 | 0 | 0 | 0 | 27 |
| ASV1453 | 0 | 0 | 0 | 0 | 0 | 23 |
| ASV1462 | 0 | 0 | 0 | 0 | 0 | 22 |
| ASV1469 | 0 | 0 | 21 | 0 | 0 | 0 |
| ASV1487 | 0 | 18 | 0 | 0 | 0 | 0 |
| ASV1537 | 0 | 0 | 15 | 0 | 0 | 0 |
| ASV1556 | 0 | 0 | 0 | 0 | 0 | 14 |
| ASV1582 | 0 | 0 | 0 | 0 | 0 | 0 |
| ASV1583 | 0 | 0 | 0 | 0 | 0 | 0 |
| ASV1667 | 7 | 0 | 0 | 0 | 0 | 0 |
| ASV1722 | 0 | 0 | 0 | 0 | 0 | 6 |
| ASV1724 | 0 | 0 | 0 | 0 | 0 | 6 |
| ASV1774 | 0 | 0 | 0 | 0 | 0 | 4 |
| ASV1809 | 0 | 0 | 0 | 0 | 3 | 0 |

Figure S1


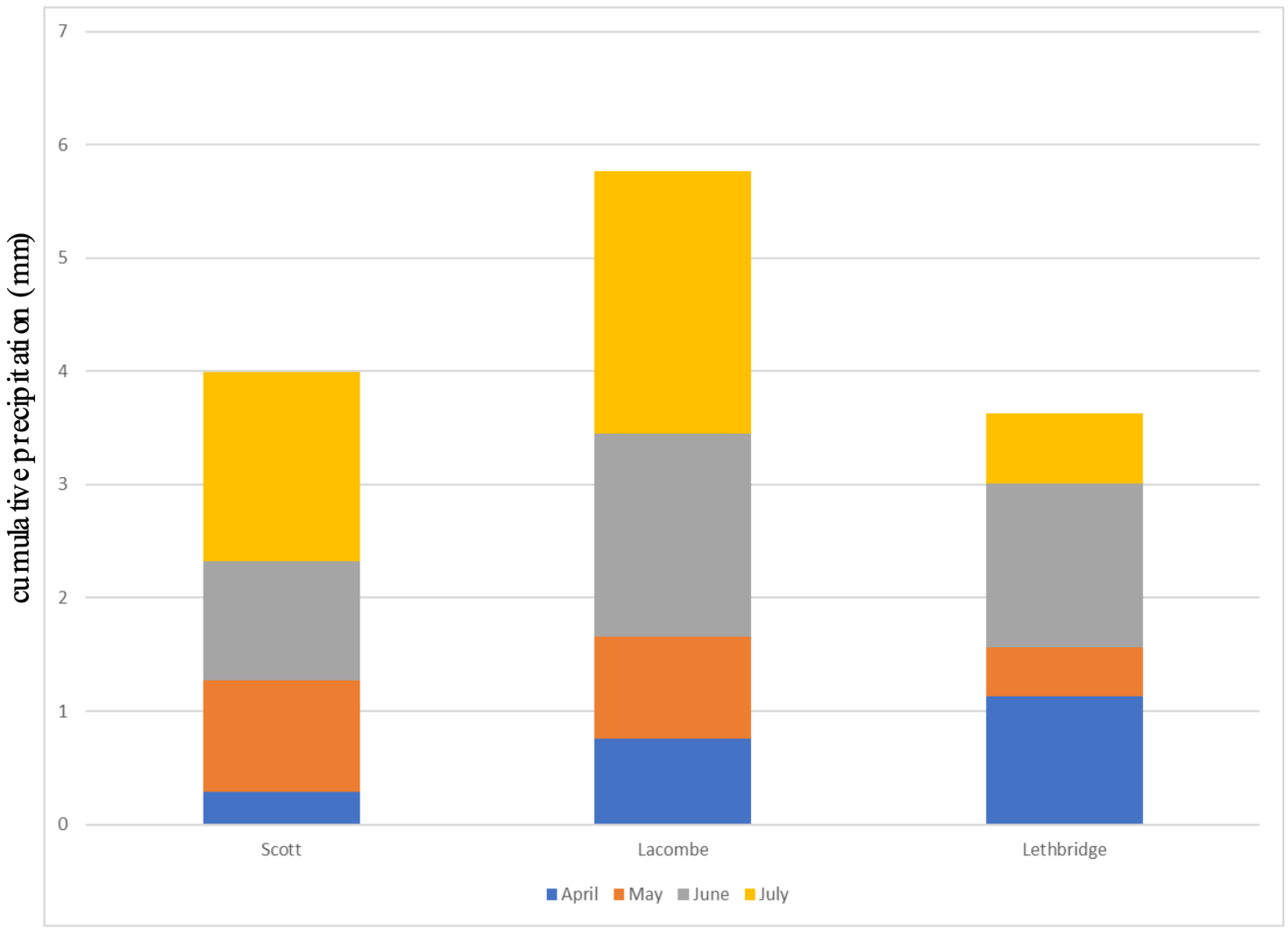


**Figure S1.**Cumulative precipitations (mm) at each site before sampling in 2018.

Figure S2


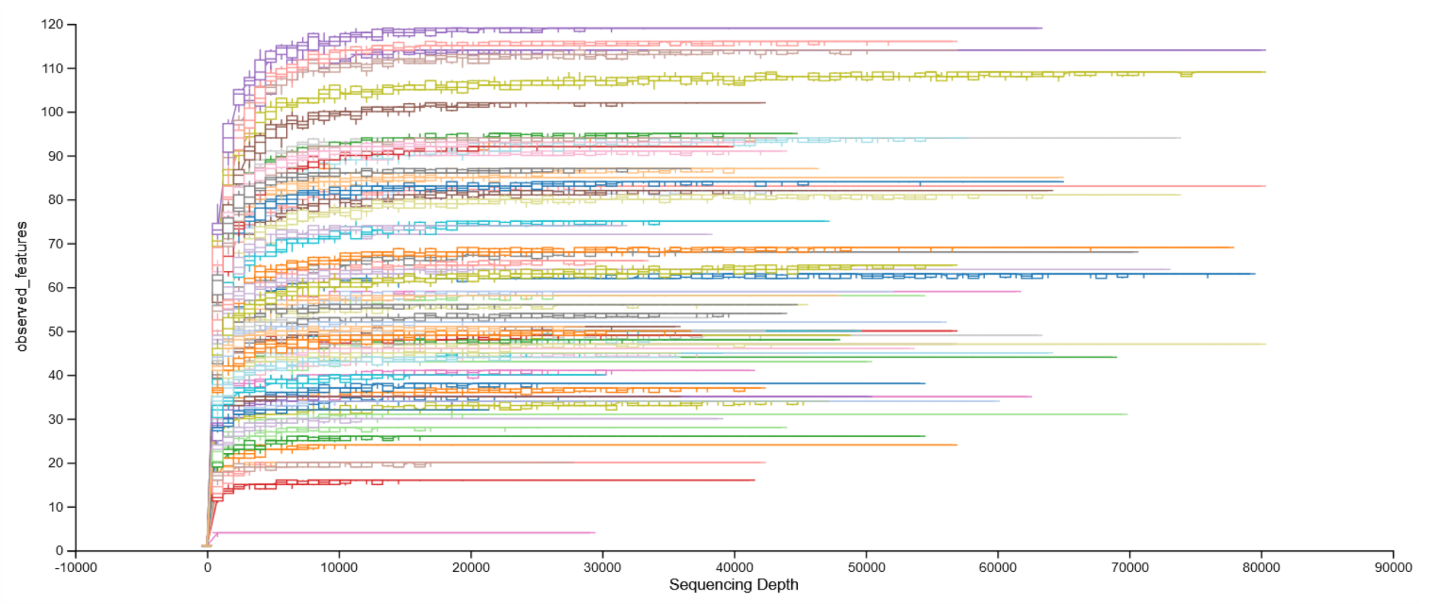


**Figure S2.** Rarefaction curves for each sample before trimming non-AMF ASV.

Figure S3

**
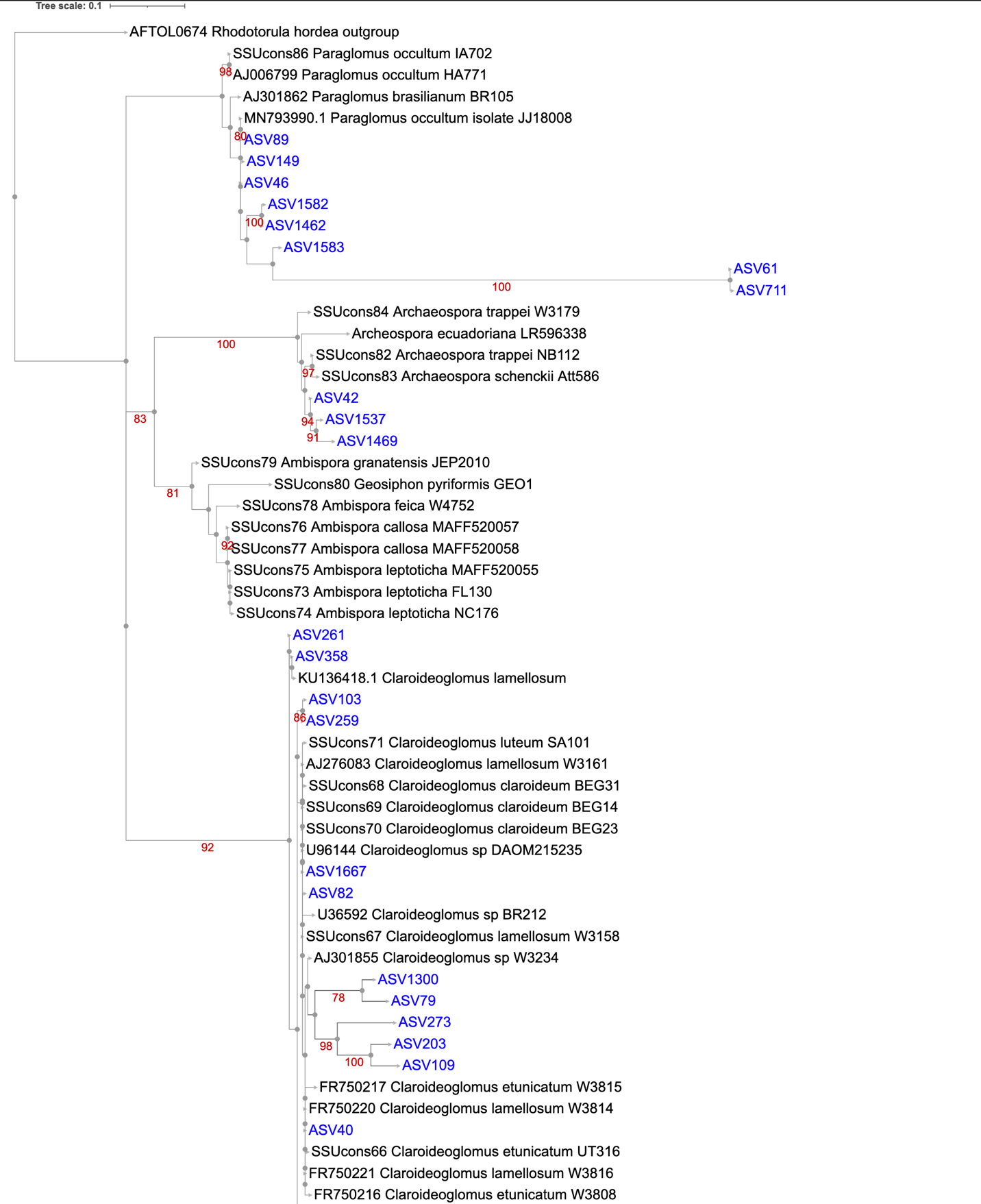
**

**
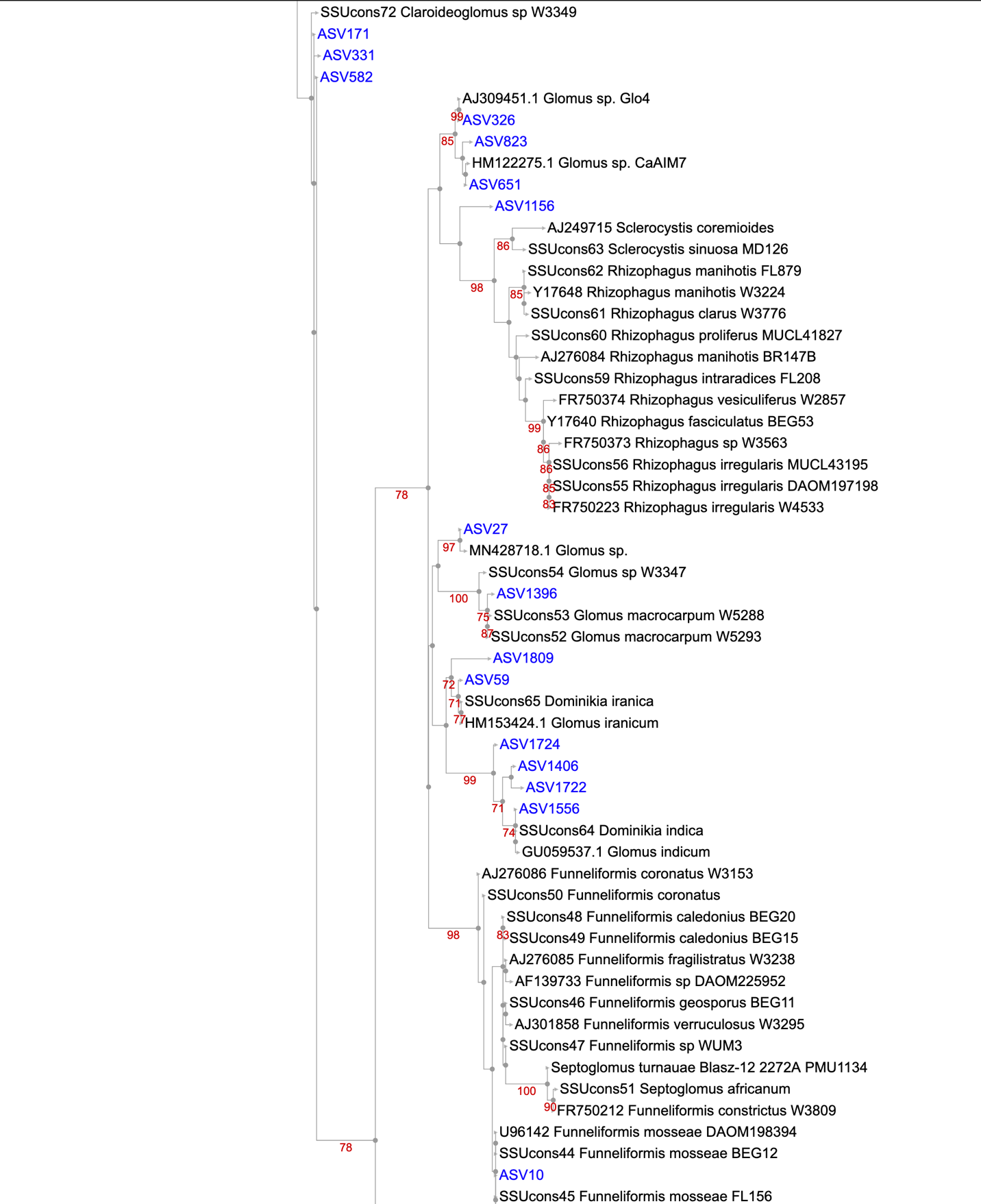

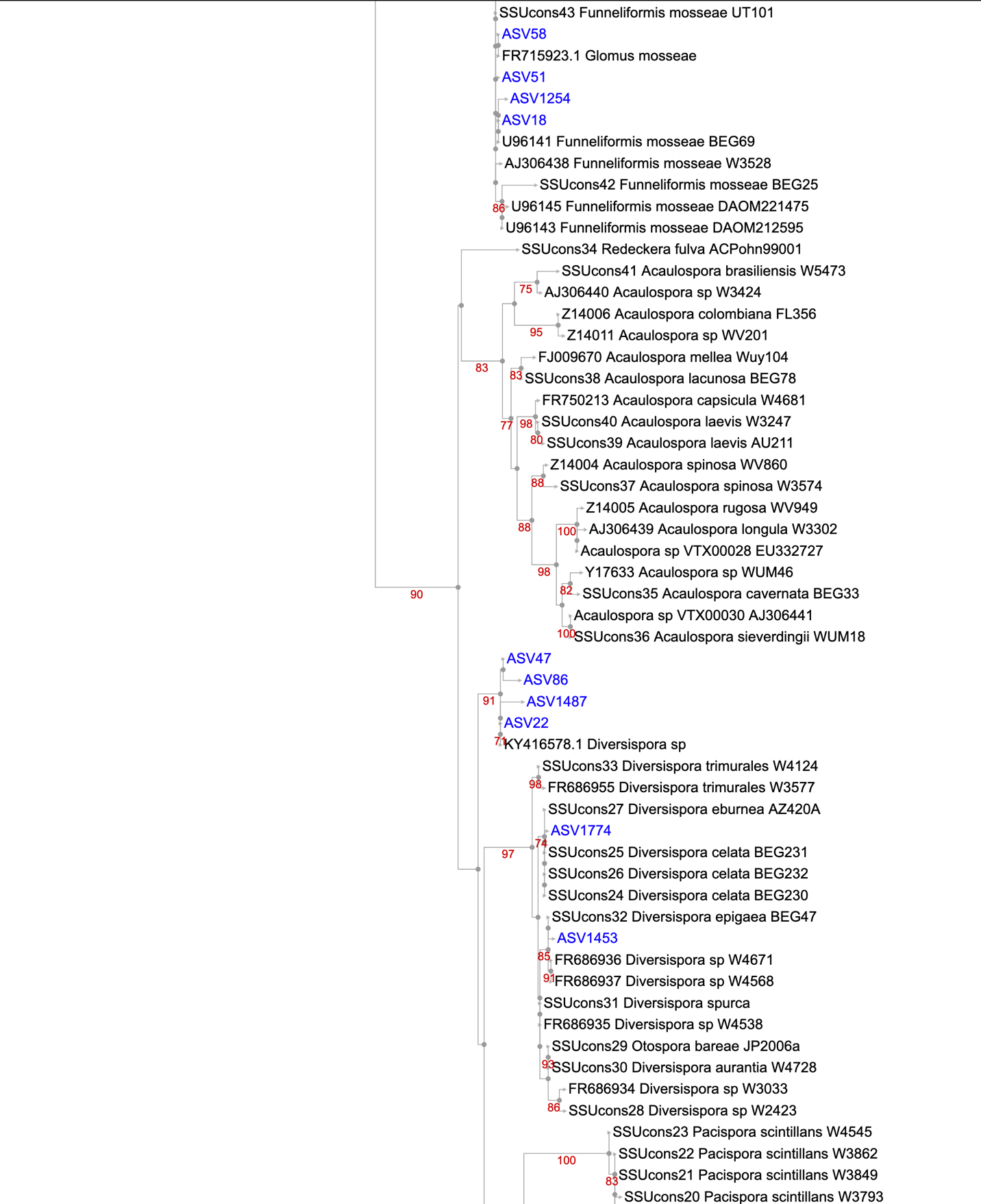

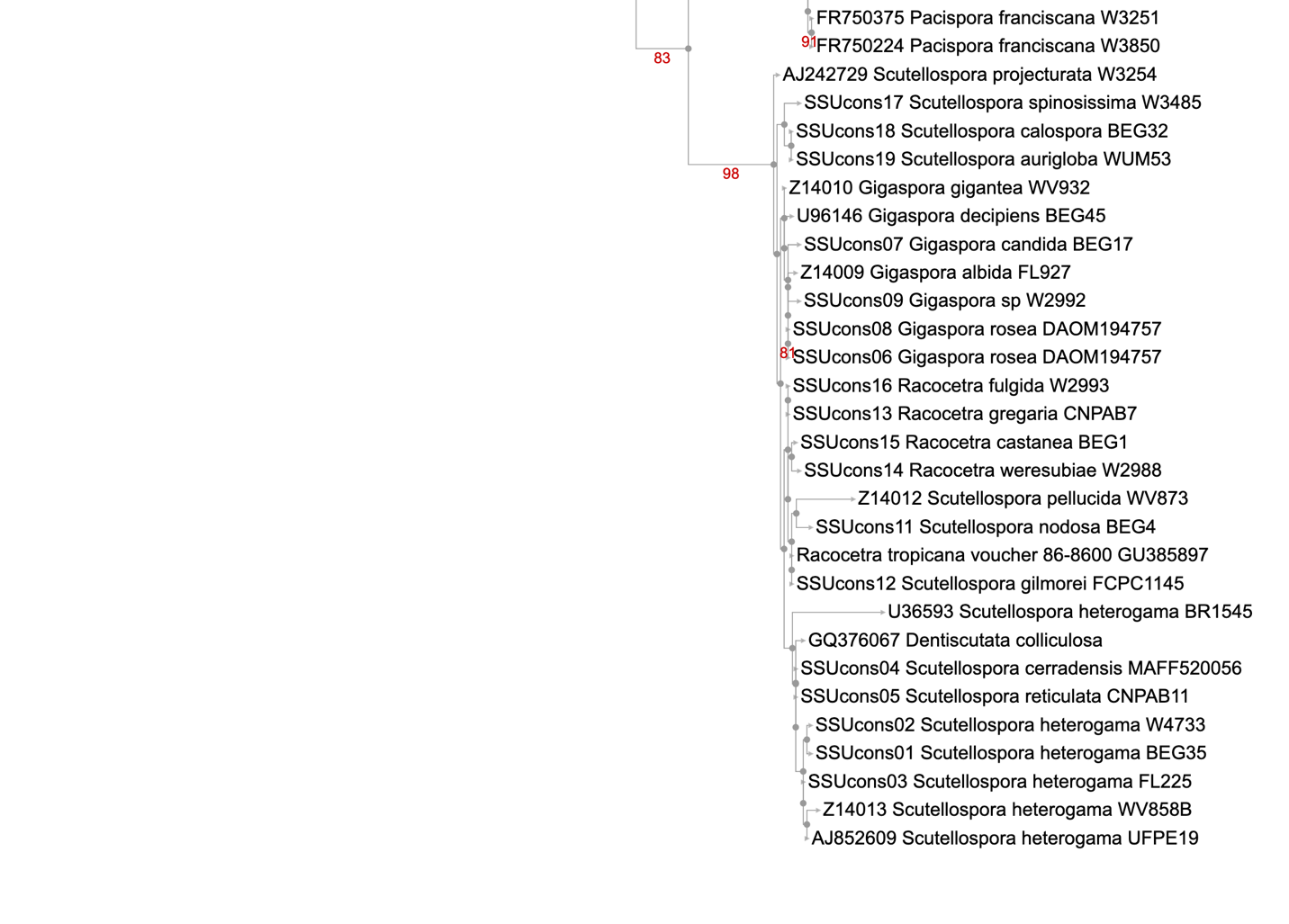
**

**Figure S3.** Phylogenetic tree of the 49 ASV identified as AMF.

Figure S4


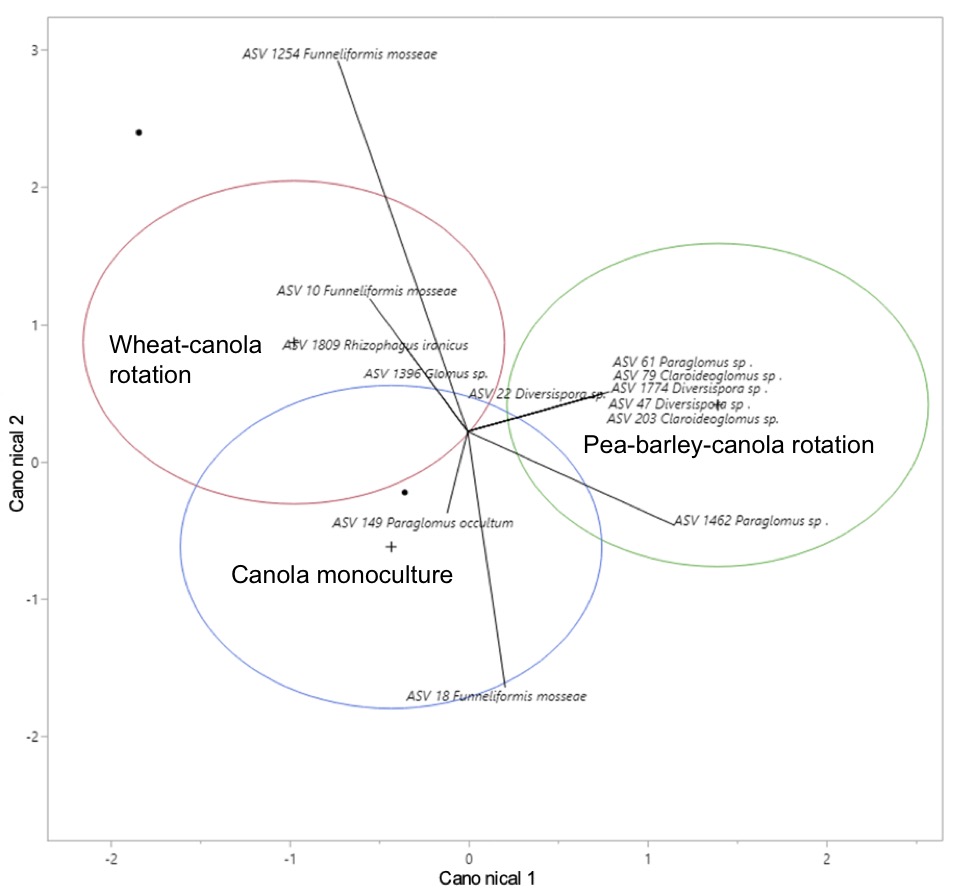


**Figure S4.**Canonical analysis of AMF community from the canola rhizosphere. Circles in red, blue and green represent different crop rotations whilevectorsshow different glomeromycotan ASV which are likely to be preferentially associated to each of the crop management. The more separated the circles are, the more the community structure between the crop management are different. The more the arrow is directed to the center of a circle, the more the ASV was associated with a certain crop rotation.
